# Taxonomic revision of the East Asian genus *Scleropteroides* Colonnelli, 1979 (Coleoptera, Curculionidae, Ceutorhynchinae)

**DOI:** 10.3897/zookeys.437.6563

**Published:** 2014-08-28

**Authors:** Junhao Huang, Hiraku Yoshitake, Runzhi Zhang, Motomi Ito

**Affiliations:** 1 CAS Key Laboratory of Zoological Systematics and Evolution, Institute of Zoology, Chinese Academy of Sciences, No. 1 Beichen West Road, Chaoyang District, Beijing 100101, China; 2 Institute of Forestry Protection, School of Forestry and Biotechnology, Zhejiang A & F University, 88 Huancheng Beilu, Linan, Hangzhou, Zhejiang 311300, China; 3 Natural Resources Inventory Center, National Institute for Agro-Environmental Sciences, 3-1-3 Kannondai, Tsukuba, Ibaraki 305-8604, Japan; 4 Department of General Systems Studies, Graduate School of Arts and Sciences, University of Tokyo, Tokyo 153-8902, Japan

**Keywords:** *Rubus*, new record, new species, *Scleropterus*, taxonomy

## Abstract

The genus *Scleropteroides* Colonnelli, 1979 (Ceutorhynchinae: Scleropterini) was revised on the basis of detailed morphological observations. The genus was redefined to include three species from East Asia: *S. hypocrita* (Hustache, 1916) is redescribed and recorded from northeastern China and northern Korea for the first time; *S. horridulus* (Voss, 1958) is redescribed with new records from southern Korea; *S. insularis* Voss, 1971 was moved from synonymy with *S. hypocrita* to that with *S. horridulus* (**syn. n.**), and *S. longiprocessus* Huang & Yoshitake, **sp. n.** is described as new, sympatric with *S. hypocrita* in Japan. All the species are associated with woody *Rubus* species (Rosaceae). A key to species, habitus photographs, illustrations of important characters, and distribution maps are provided for each species.

## Introduction

*Scleropteroides* Colonnelli, 1979 is placed in the tribe Scleropterini, subfamily Ceutorhynchinae, and is characterized mainly by a rostrum expanded from the base to apex, a visible scutellar shield, subtriangular elytra, strongly convex humeri, acute squamate granules on the elytral intervals, a deep rostral channel extending to the metaventrite, and dentate femora (Colonnelli 1979, 2004; Korotyaev 1996). This genus is thought to be related to *Scleropterus* Schoenherr, 1825 (Colonnelli 1979, Korotyaev 1996). The affinity of the two genera was supported by a recent molecular phylogenetic study (Kato et al. 2006).

The genus *Scleropteroides* was established for *Ceuthorrhynchidius hypocrita* Hustache, 1916 from Japan (Colonnelli 1979). After that, Morimoto (1984) treated *Rhytidosomus insulare* Voss, 1971 (an original incorrect spelling of “*insularis*”), which was described from the Ryukyus, as a member of *Scleropteroides*. Kim et al. (1991) and Hong et al. (1999, 2000) recorded *Scleropteroides hypocrita* from South Korea. Later, *Rhytidosomus insularis* was synonymized with *Scleropteroides hypocrita* by Colonnelli (2004) in his world catalog of Ceutorhynchinae, although Colonnelli did not provide any explanation of this taxonomic treatment. Recently, Korotyaev et al. (2014) combined *Homorosoma horridulum* Voss, 1958 with *Scleropteroides* and recorded it from Taiwan. Thus, until the present paper, *Scleropteroides* comprises *Scleropteroides hypocrita*, which is known to occur in South Korea and Japan (Morimoto 1984, 1989; Korotyaev and Hong 2004; Yoshitake et al. 2004) and to be associated with *Rubus* species (Rosaceae) (Morimoto 1984, Hong et al. 1999, Korotyaev and Hong 2004), and *Scleropteroides horridulus*, which is known to occur in Fujian Province of the continental China, Taiwan, and Japan (Korotyaev et al. 2014).

Presently, *Scleropteroides* is in need of revision. Apart from morphological differences associated with hind-wing reduction in *Scleropterus*, the distinction between the two genera is still insufficient because more than half of the defining characteristics of *Scleropteroides* are common to *Scleropterus* or even incorrectly described. Moreover, the taxonomic status of *Rhytidosomus insularis* should be revised because it shows remarkable differences from *Scleropteroides hypocrita* despite Colonnelli’s synonymy. Korotyaev et al. (2014) point out a similarity in general appearance between *Scleropteroides horridulus* and *Scleropteroides insularis*. In addition, our preliminary study suggested the presence of a new species in Japan. Finally, fundamental ecological data on *Scleropteroides* weevils are still needed.

In this study, we revise the genus *Scleropteroides* on the basis of detailed examinations of specimens collected from various localities in East Asia. In addition, we provide distributional and ecological information on *Scleropteroides* species. The systematic position of the genus, relationships among species, and the geographic distribution and host plant association of each species are discussed.

## Materials and methods

Specimens preserved in the following institutions and private collections were examined for this study: Canadian Museum of Nature, Ottawa/Gatineau (CMN); Entomological Laboratory, Faculty of Agriculture, Kyushu University, Fukuoka, Japan (ELKU); Laboratory of Environmental Entomology, Faculty of Agriculture, Ehime University, Matsuyama, Japan (EUMJ); Institute of Zoology, Chinese Academy of Sciences, Beijing, China (IZCAS); K. Izawa collection, Tajimi, Japan (KI); Muséum National d'Histoire Naturelle, Paris, France (MNHN); National Institute for Agro-Environmental Sciences, Tsukuba, Japan (NIAES); Natural History Museum Vienna, Wien, Austria (NHMV); S. Miyakawa collection, Kyushu University Museum, Fukuoka, Japan (SM); and Y. Shiozaki collection, Kawasaki, Japan (YS). All the descriptive work in this study was completed by J. Huang and H. Yoshitake.

External structures were observed under a Leica MZ95 stereoscopic microscope. Measurements of body parts are defined and abbreviated as follows: LB = body length, from the apex of the pronotum to the apices of elytra; LR = rostrum length, from a lateral view; WP = maximum width of pronotum; LP = pronotum length, from the base to the apex along the midline; WE = maximum width of elytra; and LE = length of elytra, from the base of humeri to the apex of elytra. All measurements are in millimeters. Habitus photographs were taken using a Nikon Coolpix995 digital camera attached to a Nikon SMZ1500 stereoscopic microscope. To examine terminalia, the specimens were macerated in hot water and dissected under a stereoscopic microscope. The abdomen was removed from the body and then cleaned in hot 10% KOH solution for 5–10 min. Terminalia were extracted from the abdomen and mounted on slides with glycerol (male) or pure water (female), examined using a Nikon Eclipse 55i optical microscope, and illustrated in detail using a camera lucida. Scale bars were calibrated using a Nikon objective micrometer. Details of some external structures were observed with a scanning electron microscope (Hitachi 3000-N). Plant nomenclature follows Yonekura and Kajita (2003). Distribution maps (Figs 133–134) are based on records of *Scleropteroides hypocrita* from Korea (Hong et al. 1999, 2000, Korotyaev and Hong 2004) and all the specimens examined in this study. Verbatim label data indicated by quotation marks are provided for the holotypes. Label breaks are indicated by a slash (“/”).

## Taxonomy

### Scleropteroides

Taxon classificationAnimaliaColeopteraCurculionidae

Colonnelli, 1979

Figs 7–21

Scleropteroides Colonnelli, 1979: 214. – Morimoto 1984: 316; 1989: 514 (in checklist). – Kim et al. 1991: 183 (record from Korea). – Korotyaev 1996: 459 (in key). – Hong et al. 1999: 63. – Hong et al. 2000: 123. – Yoshitake et al. 2004: 105 (in catalog). – Korotyaev and Hong 2004: 146. – Colonnelli 2004: 22, 34 (in catalog). – Korotyaev et al. 2014: 99.

#### Type species.

*Ceuthorrhynchidius hypocrita* Hustache, 1916.

#### Diagnosis.

This genus is very similar to *Scleropterus* in having a six-segmented antennal funicle, elytra bearing acute squamate granules (usually in a row on each interval), and a rostral channel extending to the metaventrite. However, it is easily distinguishable from *Scleropterus* mainly by the bisinuate basal margin of the pronotum (Figs 10, 34, 36, 38), the well-developed scutellar shield (Figs 11, 34–39), prominent elytral humeri (Figs 11, 35, 37, 39), even elytral intervals (Figs 11, 35, 37, 39), dentate femora (Fig. 15), and simple fore tibiae that are not incurved apically (Fig. 15).

#### Male.

Dark brown in general appearance. Head, rostrum, prothorax (except apical part), venter, and pygidium black; antennae, apical part of prothorax, and tarsi paler.

*Vestiture*. Body surface evenly covered with ochreous pollinosity in life. Head mainly covered with brown clavate scales, mixed with white scales; vertex with scales directed medially; forehead with scales directed basally; basal margin and median carina fringed with white ovate recumbent scales. Rostrum covered with clavate scales on basal 2/3; scales directed basally, gradually becoming smaller toward apex, replaced by hair-like scales on apical 1/3. Prothorax mainly covered with very similar scales as those on head, with longitudinal stripe of white ovate recumbent scales on median and lateral parts; each scale directed basally. Elytra (Fig. 11) bearing row of white and brown clavate scales on each interval; scales directed apically. Legs (Fig. 15) densely covered with white and brown scales; femora mostly covered with clavate semirecumbent scales, mingled with feather-like scales along inner margin; tibiae mainly covered with clavate scales, except semirecumbent hair-like scales along inner margin; corbel of each tibia fringed with brown setae. Lateral pieces of meso- and metathorax mainly covered with white ovate recumbent scales. Sterna mainly covered with white lanceolate to ovate scales; meso- and metaventral receptacles densely covered with white aciculate scales. Venter mainly covered with white lanceolate to ovate recumbent scales, mingled with white and brown clavate scales; ventrites III and IV nearly naked on disc, with only transverse row of clavate scales; ventrite V bearing fine brown scales in median concavity and white ovate scales on sides. Pygidium mainly sparsely covered with short clavate semirecumbent scales, mingled with hair-like recumbent scales; scales brown, directed ventrally.

*Head* (Figs 7–8) reticulately punctured; each puncture moderate in size; vertex with median carina from base to apex; carina becoming obscure apically; forehead shallowly depressed, with apex slightly broader than base of rostrum and then strongly widened basally; eyes medium-sized, rounded triangular, weakly convex. Rostrum (Figs 7–8) slender, evenly curved; dorsum densely rugosely punctured except apex; punctures moderate in size from base to level between antennal insertions, then becoming smaller, sparser, and shallower toward apex; sides subparallel in basal half, more or less widened before antennal insertions, then subparallel in apical part; antennal scrobes well separated on ventral surface. Antennae (Figs 8–9) inserted before middle of rostrum; scape moderate in length, evidently clavate, round and fringed with 3–4 setae at apex, slightly shorter than funicle; funicle six-segmented; club lanceolate, finely pubescent except basal part.

*Pronotum* (Figs 10–13) slightly wider than long; dorsum densely coarsely punctured, simple, lacking tubercle or prominence; punctures smaller in apical and basal parts; basal margin bisinuate, smooth, not serrate; apical margin weakly raised, with shallow median incision. Scutellar shield subovate.

*Elytra* (Figs 11–12) subcordate, nearly as long as wide, widest just behind humeri; suture evidently bent leftward; interval I of right elytron slightly broader than that of left elytron; all intervals subequal in width and height, nearly three times as wide as striae, each with row of more or less small and acute squamate granules; striae less marked, nearly naked, lacking conspicuous scales or hair, finely punctured; each puncture round, separated by distance more than three times as long as its diameter. Hind wings (Fig. 19) well-developed.

*Legs* (Fig. 15) slender; femora slightly clavate, each armed with small tooth, bearing minute squamate granules; no jumping organ present (Fig. 16); tibiae bearing minute squamate or setiferous granules; protibiae simple, lacking mucro and not curved in at apex; meso- and metatibiae moderately mucronate; corbels short, simple, not dilated outward; tarsi (Figs 17–18) moderate in length; claws free, slender, appendiculate with sharp teeth; each tooth slender, extending from base to middle of each claw.

*Underside*. Prosternum coarsely and moderately densely punctured; mesoventrite densely and finely punctured; metaventrite with dense medium-sized punctures on disc and with sparser and coarser punctures on sides; lateral pieces of meso- and metasterna sparsely coarsely punctured. Rostral channel (Fig. 13) long, extending far beyond level between posterior margins of mesocoxal cavities, with dense minute punctures; mesoventral receptacle deep, laterally costate; costae short, subparallel; metaventral receptacle very deep, terminating in steep wall and U-shaped margin; lateral walls of meso- and metaventral receptacles interrupted by inner margins of mesocoxae; metaventrite more or less prominent ventrally along apico-lateral margin of metaventral receptacle. Metendosternite as in Fig. 14. Venter coarsely and more or less densely punctured; ventrites III and IV nearly polished on disc, with only transverse row of coarse and sparse punctures; ventrite V with subtriangular median concavity along basal margin; concavity faintly sulcate along midline. Tergum as in Figs 20–21; tergite VII with pair of minute setiferous plectral tubercles near base.

*Pygidium* transverse-pentagonal, flattened, very coarsely and reticulately punctured; bottom of each puncture opaque due to dense minute punctations; upper flange arcuate downward on each side.

#### Female.

Rostrum (Figs 24–25) slightly more slender. Antennae inserted just behind middle of the rostrum. Tibiae simple, not mucronate on all legs. Ventrites I and II moderately inflated, sparsely punctured, lacking prominence. Ventrite V simple or only with longitudinal median sulcus, lacking concavity. Pygidium smaller, sectorial, mainly covered with hair-like scales. Otherwise as in male.

#### Distribution.

East Asia (China including Taiwan, Korea, and Japan; Figs 133–134).

### Scleropteroides hypocrita

Taxon classificationAnimaliaColeopteraCurculionidae

(Hustache, 1916)

Figs 1 , 2 , 22 –25 , 34 –35 , 40 – 69 , 133 , 135 –138

Ceuthorrhynchidius hypocrita Hustache, 1916: 126 (type locality: “Japon, Mont Takao, près Hachiôji”). – Chûjô 1960: 8 (Tsushima). – Nakane 1963: 374, pl.187, fig. 17 (Honshu).
Ceuthorrhynchidius hypocritus (incorrect subsequent spelling): Dalla Torre and Hustache 1930: 33 (in catalog; “Japon”). – Morimoto 1962: 194 (in checklist; Honshu, Shikoku, Kyushu).
Rhytidosomus (Rhytidosomus) holdhausi Wagner, 1944: 59 (“Süd-Japan”). – Voss 1971: 55.
Homorosoma horridulum : Chûjô and Voss 1960: 7 (in part; misidentified, not Voss 1958; Shikoku: Ehime, Omogo).
Scleropteroides hypocrita : Colonnelli, 1979: 216. – Morimoto 1985: 316, pl. 62, fig. 15 (Honshu, Shikoku, Kyushu; *Rubus* spp.); Morimoto 1989: 514 (in checklist; Honshu, Izu Islands, Shikoku, Kyushu). – Kim et al. 1991: 183 (record from Korea). – Hong et al. 1999: 63. – Hong et al. 2000: 123. – Yoshitake et al. 2004: 105 (in checklist). – Korotyaev and Hong 2004: 146. – Colonnelli 2004: 34 (in catalog).
Rhytidosomus holdhausi : Morimoto 1983: 54 (= *Scleropteroides hypocrita*).
Scleropteroides hypocritus : Morimoto 1984: 316, pl. 62, fig. 15 (Honshu, Shikoku, Kyushu; *Rubus* spp.).
Rhytidosoma (Rhytidosoma) holdhausi : Colonnelli 1994: 214 (= *Scleropteroides hypocrita*).

#### Diagnosis.

This species is characterized by the following characters: prothorax moderately constricted in the subapical part (Fig. 34); elytra with weakly prominent humeri, gently convergent toward the subapical calli (Fig. 35); scales in a row on elytral intervals, semirecumbent, evidently shorter than width of each interval (Fig. 35); apical half of the male rostrum slightly widened (Fig. 22); male metaventrite weakly prominent ventrally along the apico-lateral margin of the metaventral receptacle; male ventrite I with a median prominence along the apical margin; penis with apical projection blunt, rounded at the apex (Figs 41, 51, 61); basal part of the endophallus with a pair of fig-like sclerites (Figs 40, 50, 60); female sternite VIII with slender arms that are arcuate apically (Figs 47, 57, 67); posterior part of the bursa copulatrix densely covered with minute coniform spicules (Figs 45, 55, 65).

#### Male.

**Figures 1–6.**
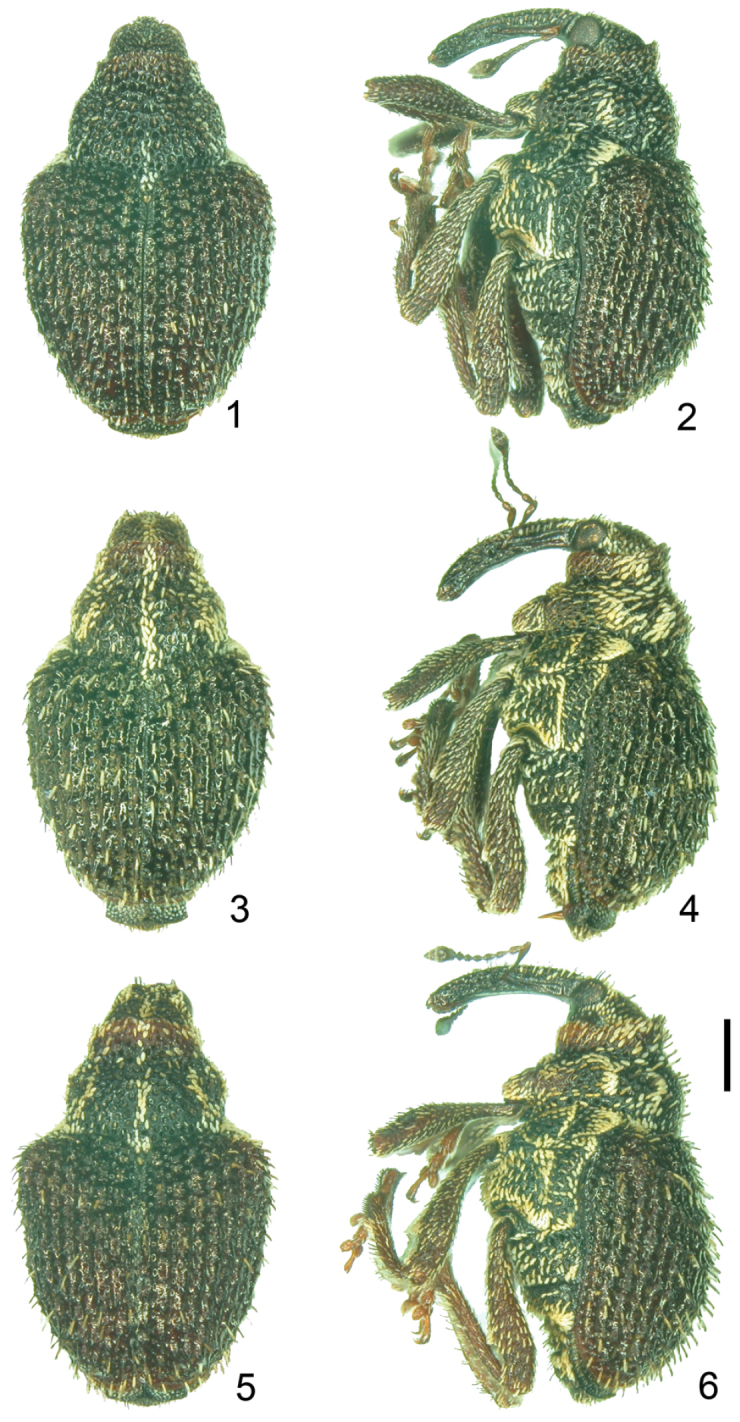
Habitus of *Scleropteroides* spp., males. **1–2** *Scleropteroides hypocrita* (Hustache) **3–4** *Scleropteroides longiprocessus* Huang & Yoshitake, sp. n. **5–6** *Scleropteroides horridulus* (Voss) **1, 3, 5** Dorsal habitus **2, 4, 6** Lateral habitus. Scale: 0.50 mm.

LB: 2.18–2.66 (mean, 2.41). LR: 0.96–1.11 (mean, 1.03). WP: 0.94–1.08 (mean, 1.01). LP: 0.78–0.97 (mean, 0.86). WE: 1.48–1.79 (mean, 1.61). LE: 1.50–1.82 (mean, 1.64). N = 10 for all measurements. Habitus as shown in Figs 1–2.

**Figures 7–21.**
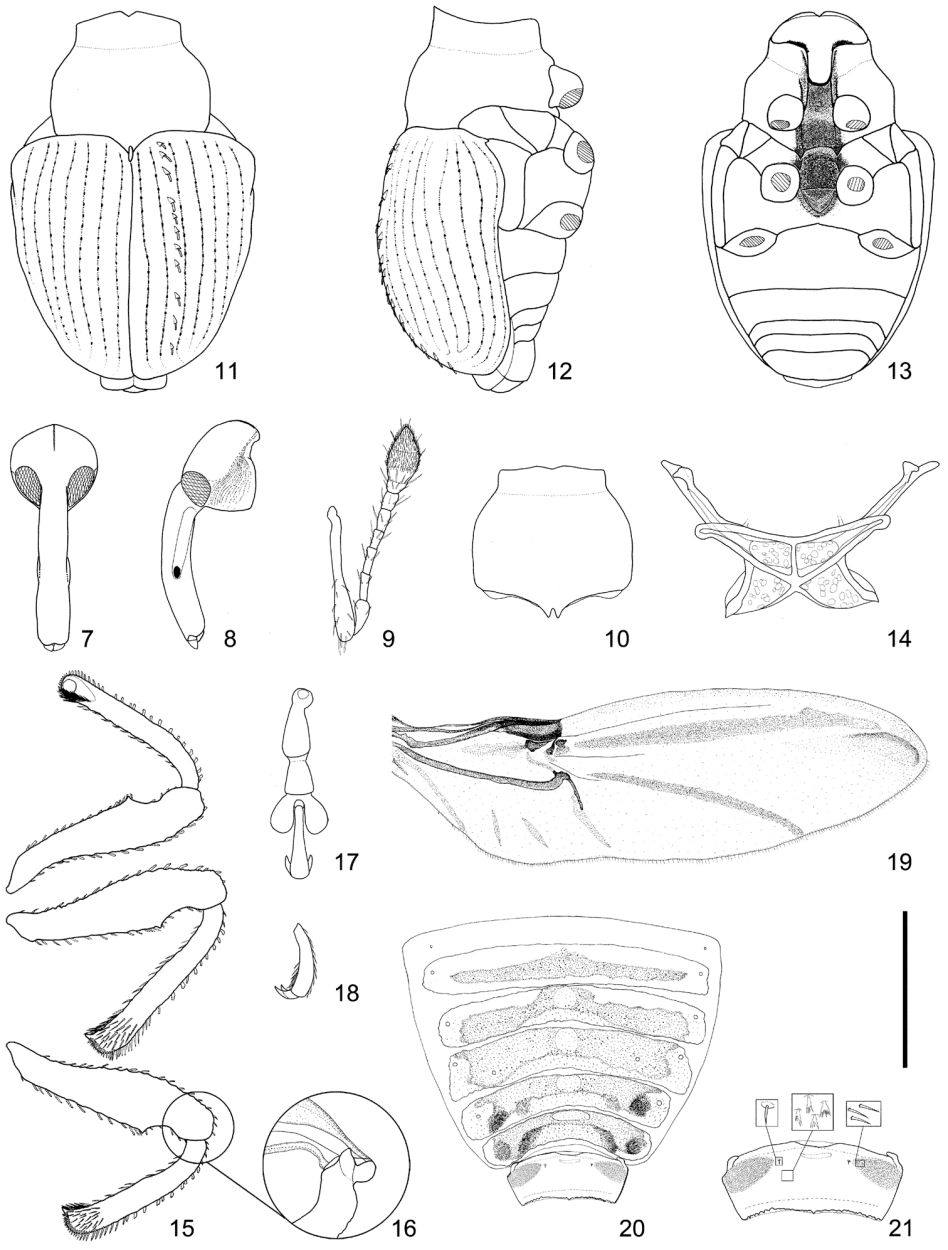
Diagnostic characteristics of *Scleropteroides*. **7** Head, dorsal view **8** Head, lateral view **9** Antenna **10** Prothorax **11** Body, dorsal view **12** Body, lateral view **13** Body, ventral view **14** Metendosternite **15** Legs **16** Base of hind-tibia, showing extensor and flexor tendons **17** Tarsus **18** Tarsomere V, lateral view **19** Hind wing **20** Tergites **21** Tergite VII. Scale: 1.00 mm for **7, 8, 10–13, 16, 19–20**; 0.75 mm for **15, 20**; 0.50 mm for **9, 14, 17–18, 21.**

*Vestiture*. Clavate scales short and semirecumbent on head, basal 2/3 of rostrum, and pronotum. Hair-like scales fine and semirecumbent on apical 1/3 of rostrum. Scales on elytral intervals (Fig. 35) semirecumbent, short, 0.4–0.8 × as long as interval width. Clavate scales on tibiae semierect.

**Figures 22–33.**
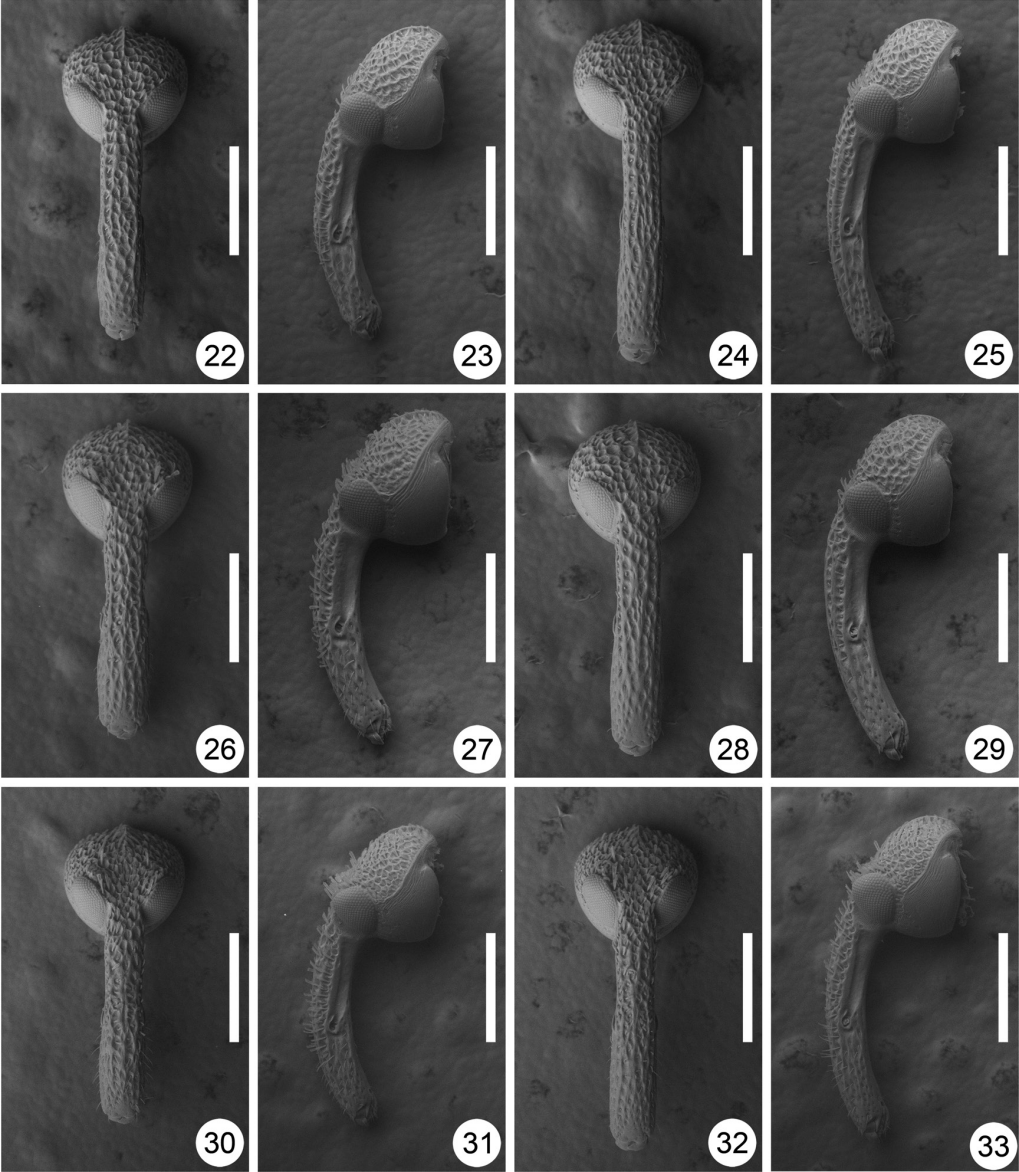
Heads of *Scleropteroides* spp. **22–25** *Scleropteroides hypocrita* (Hustache) **26–29** *Scleropteroides longiprocessus* Huang & Yoshitake, sp. n. **30–33** *Scleropteroides horridulus* (Voss) **22, 23, 26, 27, 30–31** Male **24, 25, 28, 29, 32–33** Female **22, 24, 26, 28, 30, 32** Dorsal view **23, 25, 27, 29, 31, 33** Lateral view. Scale: 0.50 mm.

*Rostrum* (Figs 22–23) slender, 1.15–1.24 × as long as pronotum; apical part of sides slightly widened and 1.14 × as long as basal part. Antennae (Fig. 23) with length ratio of funicular segments I: II: III: IV: V: VI = 1.89: 1.44: 1.36: 1.00: 1.17: 1.00 and width ratio = 1.71: 1.00: 1.21: 1.29: 1.50: 1.64.

**Figures 34–39.**
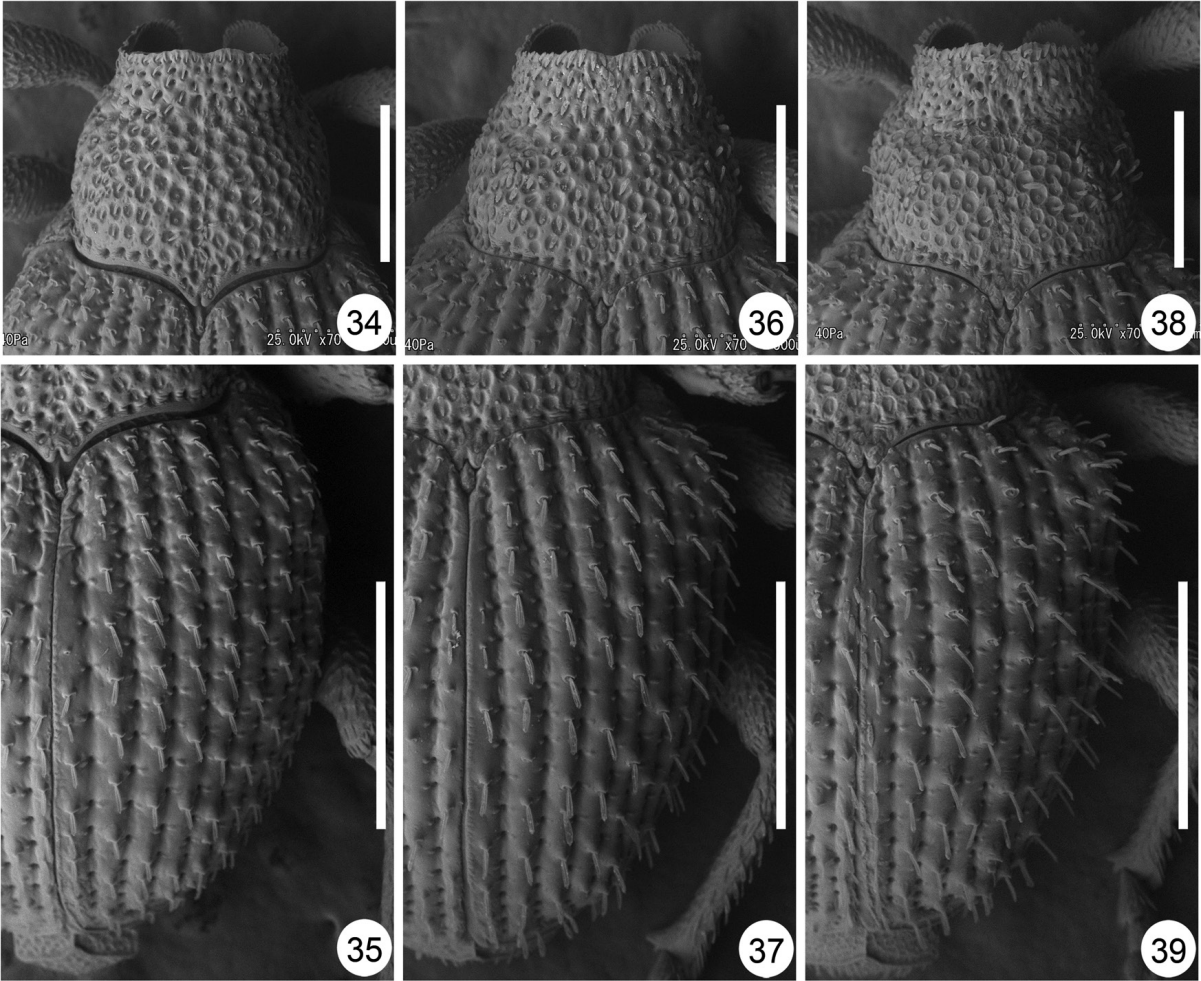
Pronotum and right elytron of *Scleropteroides* spp., male. **34–35** *Scleropteroides hypocrita* (Hustache) **36–37** *Scleropteroides longiprocessus* Huang & Yoshitake, sp. n. **38–39** *Scleropteroides horridulus* (Voss) **34, 36, 38** Pronotum **35, 37, 39** Right elytron. Scale: 0.50 mm.

*Pronotum* (Fig. 34) 1.12–1.21 × as long as wide, 0.51–0.54 × as long as and 0.60–0.66 × as wide as elytra; subapical constriction moderate; sides slightly widened from base to basal 1/3, faintly narrowed to middle, then gradually convergent toward apex.

*Elytra* 0.98–1.07 × as long as wide, 1.87–1.97 × as long as and 1.51–1.66 × as wide as pronotum, gently convergent toward subapical calli; humeral calli weakly prominent; subapical calli weakly prominent.

*Underside*. Metaventrite weakly prominent ventrally along apico-lateral margin of metaventral receptacle. Venter coarsely and moderately densely punctured; ventrite I with median prominence along apical margin; prominence weak, semicircular, densely and finely punctured; ventrite V with more or less shallow median concavity; length ratio of ventrites I: II: III: IV: V = 4.18: 2.00: 1.00: 1.14: 1.82 and width ratio = 1.96: 1.75: 1.32: 1.18: 1.00.

*Pygidium* transverse-pentagonal.

**Figures 40–49.**
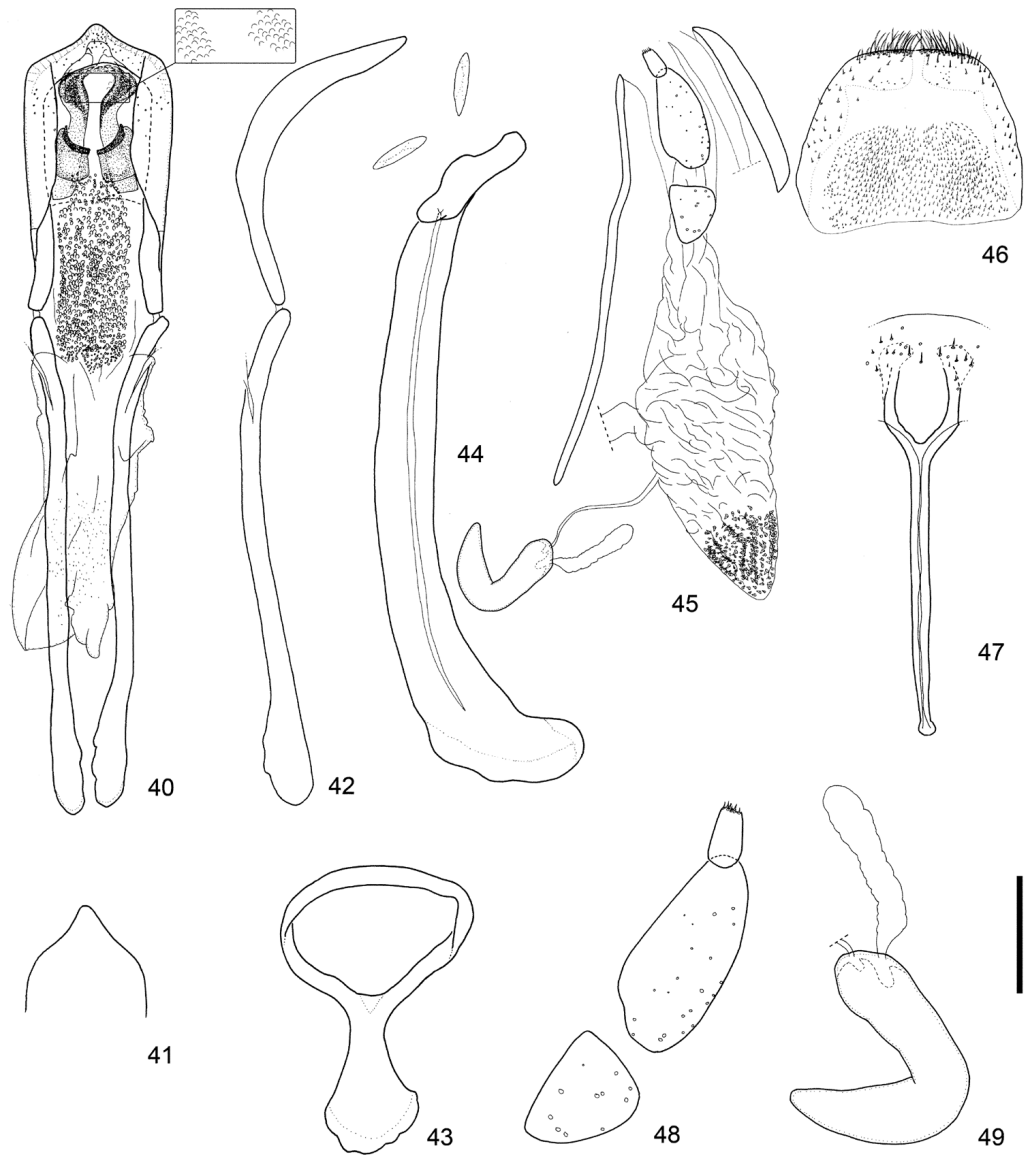
Male and female terminalia of *Scleropteroides hypocrita* (Hustache) from Takaosan, Japan. **40** Aedeagus, dorsal view **41** Apex of the penis, dorsal view **42** Aedeagus, lateral view **43** Tegmen **44** Sternites VIII and IX, male **45** Female terminalia and genitalia, lateral view. **46** Tergite VIII, female **47** Sternite VIII, female **48** Coxite and stylus. **49** Spermatheca. Scale: 0.20 mm for **40–47**, 0.10 mm for **48–49.**

**Figures 50–59.**
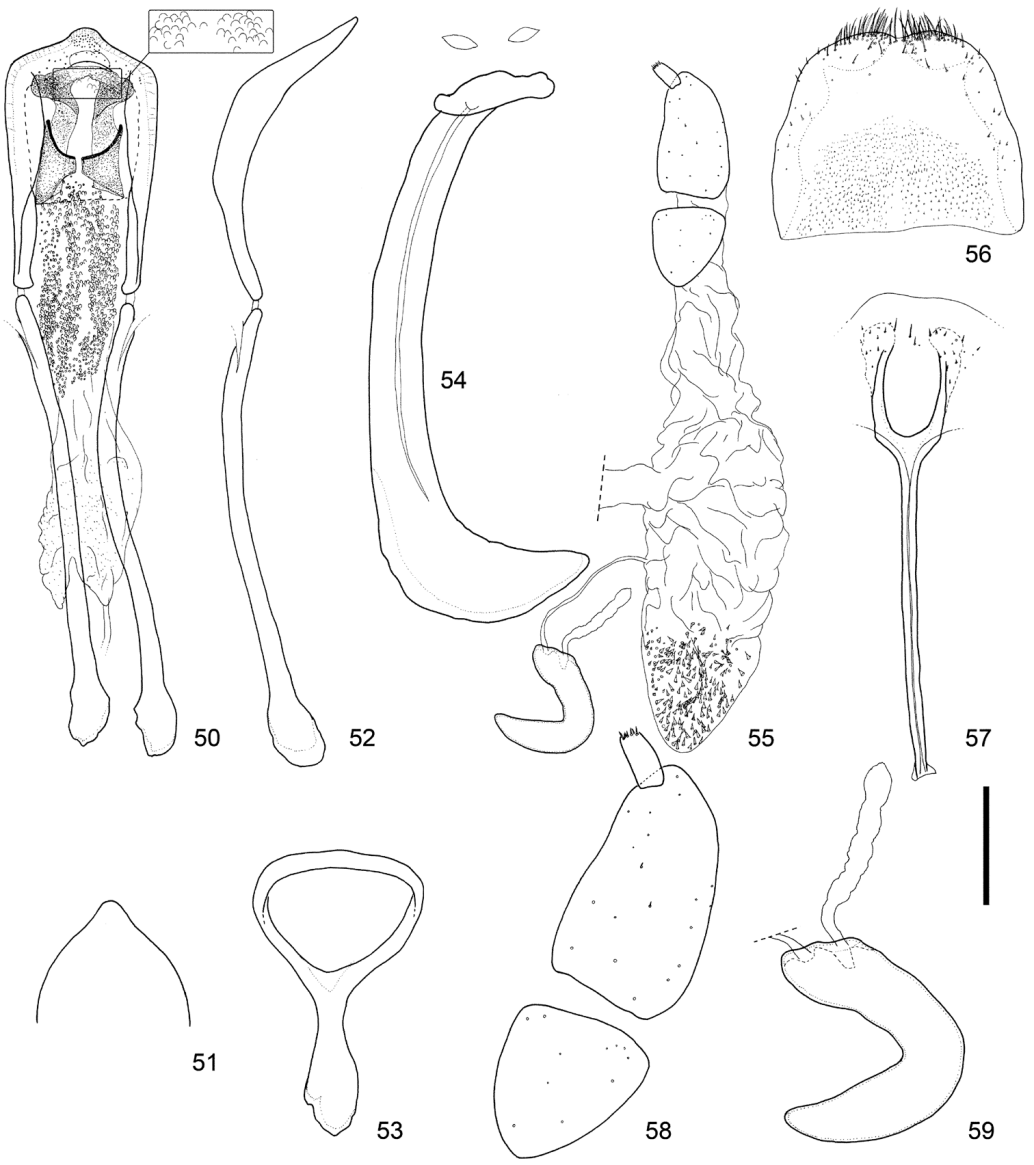
Male and female terminalia of *Scleropteroides hypocrita* (Hustache) from Mt. Jirisan, Korea. **50** Aedeagus, dorsal view **51** Apex of the penis, dorsal view **52** Aedeagus, lateral view **53** Tegmen **54** Sternites VIII and IX, male **55** Female genitalia, lateral view **56** Tergite VIII, female **57** Sternite VIII, female **58** Coxite and stylus **59** Spermatheca. Scale: 0.20 mm for **50–57**, 0.10 mm for **58–59.**

**Figures 60–69.**
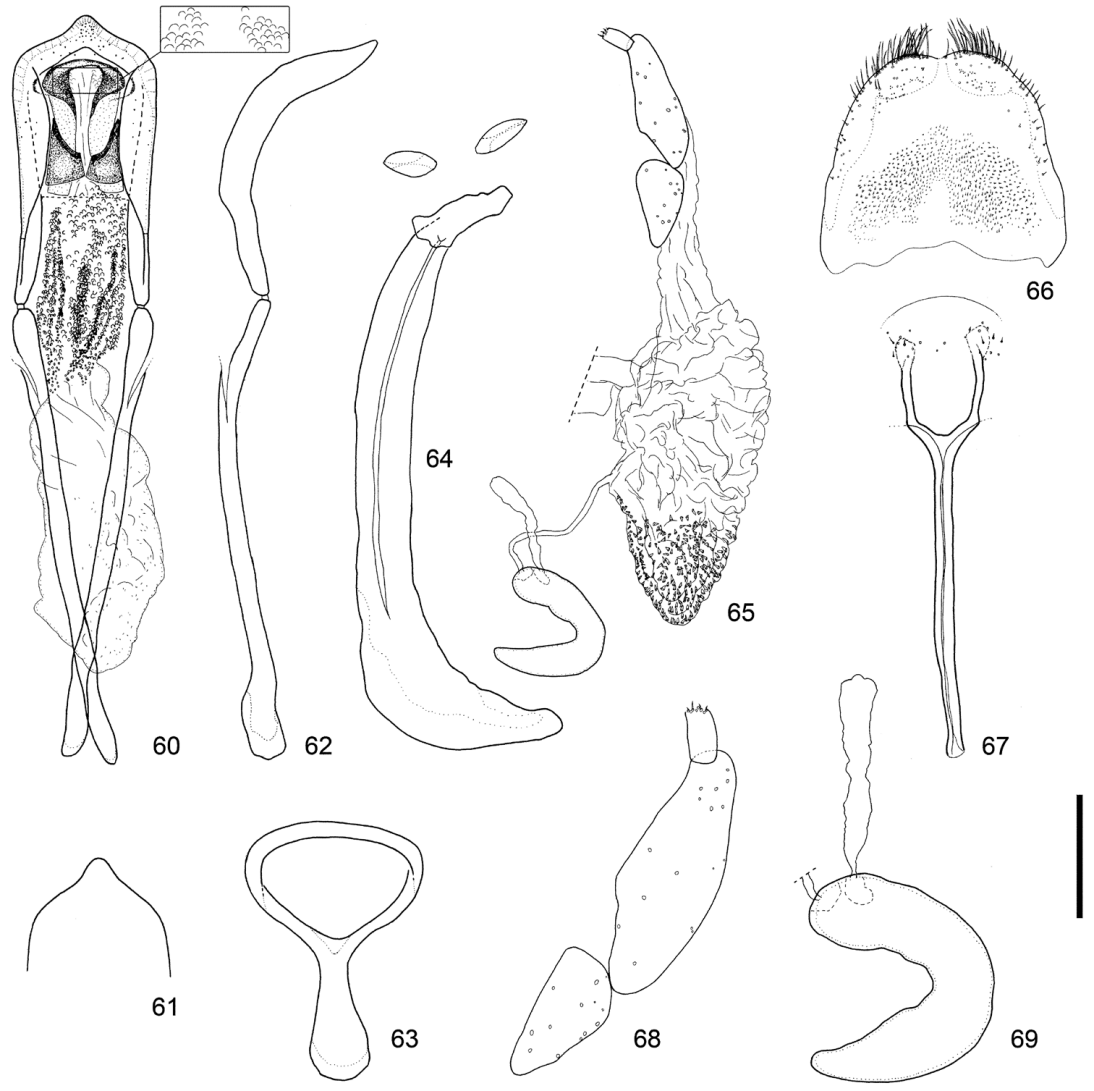
Male and female terminalia of *Scleropteroides hypocrita* (Hustache) from Heilongjiang, northeastern China. **60** Aedeagus, dorsal view **61** Apex of the penis, dorsal view **62** Aedeagus, lateral view **63** Tegmen **64** Sternites VIII and IX, male **65** Female genitalia, lateral view **66** Tergite VIII, female **67** Sternite VIII, female **68** Coxite and stylus **69** Spermatheca. Scale: 0.20 mm for **60–67**, 0.10 mm for **68–69.**

*Terminalia and genitalia*. Sternite VIII (Figs 44, 54, 64) diminished into pair of eye-shaped sclerites; spiculum gastrale robust, evidently longer than penis or its apodeme, bent leftward. Tegmen (Figs 43, 53, 63) with apodeme more or less stout, nearly 0.8 × as long as the diameter of the tegminal ring, more or less widened toward apex. Penis (Figs 40–42, 50–52, 60–62) broad, relatively thin in profile, more or less moderately curved downward in the basal 2/3, then slightly bent upward in apical 1/3; sides faintly narrowed from base to basal 1/3, slightly broadened from basal 1/3 to apical 1/5, then strongly convergent apically; apical projection blunt, rounded at apex. Basal part of endophallus (Figs 40, 50, 60) with pair of fig-like sclerites, numerous rounded spicules in median part, and moderately dense minute spicules in apical part.

#### Female.

LB: 2.28–2.67 (mean, 2.50). LR: 1.05–1.31 (mean, 1.19). WP: 0.95–1.11 (mean, 1.03). LP: 0.80–0.97 (mean, 0.89). WE: 1.54–1.89 (mean, 1.72). LE: 1.57–1.82 (mean, 1.72). N = 11 for all measurements.

*Rostrum* (Figs 24–25) slightly more slender, 1.27–1.36 × as long as pronotum.

*Pronotum* 1.11–1.20 × as wide as long.

*Elytra* 0.97–1.03 × as long as wide.

*Underside*. Metaventrite more strongly prominent ventrally along apical margin of metaventral receptacle. Ventrites I and II moderately inflated, sparsely punctured, lacking prominence. Ventrite V lacking concavity, with only longitudinal median sulcus that is sometimes obscure.

*Pygidium* smaller, fan-shaped.

*Terminalia and genitalia*. Tergite VIII (Figs 46, 56, 66) with pair of combs of dense, long setae along apical margin. Sternite VIII (Figs 47, 57, 67) with pair of patches of several minute setae near apex; arms relatively slender, nearly 0.8 × as long as apodemes, nearly half as long as coxite and stylus combined, slightly basally fused, and apically arcuate; apodemes slender, moderately divergent near apex. Bursa copulatrix (Figs 45, 55, 65) with dense minute coniform spicules in posterior part. Coxites (Figs 48, 58, 68) robust, subdivided into two pieces, nearly 7.0 × as long as styli; styli apicolaterally inserted, moderate in length, nearly 2.0 × as long as wide. Spermatheca (Figs 49, 59, 69) with cornu slender, strongly curved and slightly attenuate; collum slightly convex; ramus indistinct; insertions of duct and gland close to each other.

Otherwise as in male.

#### Material examined.

HOLOTYPE: 1 female (MNHN), “Mont Takao/près Hachiôji/Japon 30-5-08/Edme Gallois; MUSEUM PARIS/NIPPON MOYEN/E. GALLOIS 1912; *Ceuthorrhynchidius*/*hypocritus*/*Hust.*/*type*” (hand written). HOLOTYPE OF RHYTIDOSOMUS (RHYTIDOSOMUS) HOLDHAUSI WAGNER, 1944: 1 male (NHMV), "*Rorelz*/*1875*/*Süd*/*Japan*" (hand-written on grayish card); "♂" (typed on white card); "Typus" (typed on orange card); "*Hÿshichosoma*/det.H.Wagner/*Holdhausi m.*/*Tÿpe*! ♂" (hand-written in red on white card, partially typed); "TYPUS" (typed on red card); "*Holdhausi*/*Jap. Wagn.*" (hand-written on grayish card); "*Scleropteroides*/*hypocrita (Hustache, 1916)*/E. Colonnelli det. 19*91*" (hand-written on white card, partially typed). **CHINA: Liaoning.** 1 male and 1 female, Benxi, 41°17'N, 123°44'E, 3-VI-1963, H. Li (IZCAS, IOZ(E)896779 and 896770). 1 male, Anshan, Qianshan, 41°05'N, 123°06'E, 9-VI-1963, H. Li (IZCAS, IOZ(E)896773). 1 male and 1 female, Fengcheng, Tongyuanpu, 40°17'N, 123°55'E, 30-VI-1963, H. Li (IZCAS, IOZ(E)896771–896772). **Heilongjiang.** 2 males and 2 females, Ercengdianzi, 45°26'N, 127°07'E, 15–22-VI-1941 (IZCAS, IOZ(E)896474–896477). **D.P.R. KOREA: Pyonganbukdo.** 1 female, Mt. Myohyang-San, Around Habiro, 200–550 m, 29-VI- 2009, C. Han (NIAES). **Pyeongyangjikhalsi.** PyongYang-City, around Mt. RyongAk-San, near Suna-river: 1 male and 2 females, 18-V-2012, C. Han (NIAES); 1 female, 19–22-V-2012, C. Han (NIAES). **R. KOREA: Gangwondo.** 3 males and 5 females, Chuncheongun, Dongmyeon, Gamjeongri, 21-V-1992, K. Morimoto (ELKU). 3 males and 1 female, Chuncheongun, 11-VI-1997, K. Morimoto (ELKU). **Gyeonggido.** Pocheongun, Kwangnung: 2 males and 3 females, 16-V-1984, K. Morimoto (ELKU); 1 male and 1 female, 14–19-V-1992, M. T. Chujo (ELKU). **Gyeongsangbukdo.** 1 male and 1 female, Yeonggi, 14-VI-1997, K. Morimoto (ELKU). **Gyeongsangnamdo.** 1 female, Mt. Jirisan, Daesungri, 8-VI-1991, J. D. Bae (ELKU). 1 female, Mt. Jirisan, Bycjum Valley, 9-VI-1983, Lee Lab (ELKU). 1 female, Mt. Jirisan, Yeongsinbong–Samsinbong, 17-VI-1994, H. Kojima (ELKU). 1 female, Mt. Jirisan, Piagol, 30-V-2000, H. Yoshitake (NIAES). 1 male, Mt. Jirisan, Simweon, 31-V-2000, S. Kamitani (NIAES). 1 male, Mt. Jirisan, 31-V-2000, H. Yoshitake (NIAES). Hamyanggun, Macheonmyeon, Samjeongri, Mt. Jirisan: 2 males and 2 females, 9-V-1991, J. D. Bae (ELKU); 1 male, 9-V-1991, K. Morimoto (ELKU); 3 females, 11-V-1991, J. D. Bae (ELKU); 2 males and 3 females, 15-V-1991, J. D. Bae (ELKU); 4 males and 1 female, 6-VI-1991, J. D. Bae (ELKU); 2 males and 2 females, Samjeongrurak, 7-VI-1991, J. D. Bae (ELKU); 1 male, 14-VII-1991, K. Morimoto (ELKU); 13 males and 17 females, 15-V-1991, K. Morimoto (ELKU); 6 males and 6 females, 14-VI-1994, H. Kojima (NIAES); 5 females, 516 m, 35°21'09.2"N, 127°38'58.8"E, 5-V-2005, H. Yoshitake, on *Rubus crataegifolius* (NIAES). **Jeollabukdo.** 1 male and 1 female, Namwongun, Manbok Valley, 1-VI-1991, J. D. Bae (ELKU). 1 male and 5 females, Namwongun, Deongdong Valley, 7-VI-1991, J. D. Bae (ELKU). 1 male, Namwongun, Nogodan, 12-VII-1991, J. D. Bae (ELKU). 3 males and 1 female, Namwongun, Deongdongri, 19-VI-1994, H. Kojima (ELKU). Namwongun, Sannaemyeon, Simwon Valley: 1 female, 10-V-1991, K. Morimoto (ELKU); 1 male and 3 females, 13-V-1991, J. D. Bae (ELKU). Namwongun, Sannaemyeon, Jeonglyongchy: 2 females, 12-V-1991, K. Morimoto (ELKU); 1 male and 1 female, 14-V-1991, K. Morimoto (ELKU); 1 male and 1 female, 5-VI-1991, J. D. Bae; 1 male, 12-VI-1991, J. D. Bae (ELKU). **JAPAN: Hokkaido.** 1 male, Hidaka, 11-VII-83, Y. Shiozaki (YS). 8 males and 4 females, Taisei, Hirahama, 7-VI-2003, T. Miyata (NIAES). 1 male and 1 female, Kamiiso, Tomigawa, Hosokomatazawa, 27-VI-1993, T. Miyata (NIAES). 1 female, Fukushima, Mitake, 13-VI-1998, H. Yoshitake (NIAES). 1 male and 2 females, Assabuchou, Shimizu, 14-VI-1998, H. Yoshitake (NIAES). **Honshu.** AOMORI. Shimokita-hantou Peninsula, Oohata, Yunomata: 1 female, 16-VII-1956, K. Morimoto (ELKU); 1 female, 26-VII-1956, K. Morimoto (ELKU). 1 male and 1 female, Misawa, Yachigashira, 7-VI-2007, K. Morimoto (NIAES). 1 male, Hachinohe, Tanesashi-kaigan, 10-VI-2007, K. Morimoto (NIAES). IWATE. 1 male and 2 females, Miyako, Kamegamori, 15-VI-1986, K. Emoto (NIAES). 2 females, Sawauchi, Yasugasawa, 31-V-1998, H. Yoshitake (NIAES). 2 males and 1 female, Hanamaki, Toyosawa-rindou, 338–313 m, 39°28'36.2"–39°29'08.6"N, 140°56'15.7"–140°56'32.9"E, 8-VI-2007, H. Yoshitake, on *R. crataegifolius* (NIAES). 1 male and 1 female, Kawai, Yoshibezawa, 9-VI-2007, K. Morimoto (NIAES). 1 male, Kuji, Ootsuki-touge Pass, 10-VI-2007, K. Morimoto (NIAES). 1 male and 3 females, Kawai, Tsuchisaka-touge Pass, 12-VI-2007, J. Kantoh (NIAES). 1 male, Matsukusa, 800 m, 21-VI-1989, M. J. Sharkey (CMN). 3 males and 2 females, “Mt. Hyacinthe” (most probably an error in writing Mt. Hayachine), 500 m, 21-VI-1989, M. J. Sharkey (CMN). MIYAGI. 1 male and 3 females, Marumori, Fudou, 5-V-1994, H. Yoshitake (NIAES). 1 female, Shichigashuku, Inago, 20-V-1994, H. Yoshitake (NIAES). 1 female, Shiroishi, Mizubashounomori, 27-VI-1994, H. Yoshitake (NIAES). AKITA. Lake Tazawako: 2 females, 4-VI-1969, S. Miyakawa (SM); 3 males, 12-VI-1971, S. Miyakawa (SM); 1 male and 1 female, 15-VI-1971, S. Miyakawa (SM). 3 males and 2 females, Karabuki-shitsugen, Kuroyu, 12-VI-1974, S. Miyakawa (SM). 2 females, Hiyagata–Kunimi-onsen, 16-VI-1974, S. Miyakawa (SM). 3 males and 2 females, Senboku, Nishiki, Ainai, 10-VI-1983, S. Miyakawa (SM). YAMAGATA. 1 female, Yonezawa, Kariyasu, 15-VI-1975, S. Miyakawa (SM). 5 males and 4 females, Mt. Zaousan, Yoshikari-rindou, 18-VI-1983, S. Miyakawa (SM). 1 male, Oguni, Kotamagawa, 300 m, 6-VI-2005, H. Hirano (NIAES). 1 female, Oguni, Tamagawa, 120–150 m, 6-VI-2005, H. Hirano (NIAES). FUKUSHIMA. 1 female, Kamitoriwata, 20-VI-1976 (NIAES). Iwaki, Eda: 2 males and 1 female, 25-V-1980, Y. Shiozaki (YS). 3 males and 3 females, Minamiaizu, Funamata-rindou, 20-V-1983, S. Miyakawa (SM). 2 males, Namie, 30-IV-1994, H. Yoshitake (NIAES). Haranomachi, Yokokawa, Akane-rindou: 1 male, 15-VI-1984, K. Kinugasa (NIAES); 1 male, 31-V-1987, H. Ebihara (NIAES). Haranomachi, Kozikiishi-rindou: 11 males and 28 females, 4-VI-1988, S. Miyakawa (SM); 1 male, 4-VI-1988, S. Miyakawa (NIAES); 14 males and 9 females, 5-VI-1988, S. Miyakawa (SM). Hinoemata: 1 male, 14-VI-1980, Y. Shiozaki (YS); 1 female, 20-VI-1990, H. Kojima (ELKU); 2 females, Mikawa-rindou, 24-VI-1990, K. Yoshihara (SM). 1 male and 1 female, Nishigou, Yukiuribashi, 3-V-2002, S. Mizunoya (NIAES). 1 female, Nishigou, Daijou, 22-V-2002, S. Mizunoya (NIAES). Nishigou, Mabune: 1 male and 2 females, 650 m, 3-VI-2005 (NIAES), H. Hirano; 1 male and 1 female, 770 m, 3-VI-2005, H. Hirano (NIAES); 6 males and 8 females, 3-VI-2005, H. Yoshitake, on *Rubus* sp. (NIAES). IBARAKI. 3 males and 7 females, Mt. Yamizosan, Shimokitazawa, 28-V-1988, Y. Kurosawa (SM). 1 female, Takahagi, 29-V-1996, H. Takizawa (NIAES). TOCHIGI. 1 female, Mt. Sukaizan, 4–5-VII-1971, H. Takizawa (NIAES). 1 male and 1 female, Nishinasuno, 8-V-1977, S. Miyakawa (SM). 4 males and 3 females, Nikko, Kashiwagi, 16-V-1982, S. Miyakawa (SM). 1 male, Nikko, Nebazawadani, 19-VII-1940, S. Miyakawa (SM). 1 male, Fujiwara, Midorizawa-rindou, 21-V-1989, S. Miyakawa (SM). 1 female, Nasu, Toyahara, 13-VII-1996, H. Takizawa (NIAES). 1 female, Nasu, Mt. Minamigassan, 20-VII-1996, H. Takizawa (NIAES). 2 males and 1 female, Nasu-kougen, 1200–1500 m, 7-VI-2005, H. Hirano (NIAES). 4 males and 3 females, Nishinasuno, N. G. R. I., 500 m, 10-VIII-1989, M. J. Sharkey (CMN). GUNMA. 1 male and 1 female, Utsunomiya, 6-V-1968, H. Takizawa (NIAES). 2 females, Mt. Hotakayama, 13–15-VII-1975, H. Irie (ELKU). 1 female, Akagi, 21-V-1967 (SM). 1 male, Akagi, Akagi-shizenen, 9–10-V-1988, S. Saito (NIAES). 1 female, Akagi, Mt. Akagisan, 3-VI-1997, T. Ishikawa (NIAES). 1 male, Oku-tone, Lake Fujiwarako, 13-V-1990, S. Tsuyuki (NIAES). 4 males and 5 females, Niiharu, Amemi-rindou, 4-V-1998, S. Arai (NIAES). 5 males and 5 females, near Mt. Aneyama, 19-V-2001, S. Arai (NIAES). Matsuida, Kirizumi-onsen: 1 female, 27-V-1989, K. Matsumoto (NIAES); 1 male and 1 female, 27-V-1989, H. Kojima (NIAES); 1 male and 1 female, 17-V-1998, S. Arai (NIAES). SAITAMA. 2 males and 2 females, Shoumaru-touge Pass, 9-V-1976, S. Miyakawa (SM). 1 male, Minano, Shimohinozawa, Sawabe, 1-VI-1984, S. Miyakawa (SM). 1 female, Ootaki, near Taiyouji, 4-VII-1999, S. Arai (NIAES). CHIBA. 4 males and 2 females, Mt. Kiyosumiyama–Kenminnomori, 31-III-1979, J. Okuma (SM). 1 female, Bousou-hantou Peninsula, Mt. Kiyosumiyama, 30-IV-1991, S. Tsuyuki (NIAES). TOKYO. 1 male, Mitakadai, 30-V-1941, S. Miyakawa (SM). 1 male and 1 female, Mitaka, 25-V-1948, S. Miyakawa (SM). Mt. Oodakeyama: 1 male and 2 females, 21-V-1942, S. Miyakawa (SM); 4 males and 2 females, 24-V-1942, S. Miyakawa (SM). 1 female, Mt. Mitakesan, 15-V-1966 (SM). 1 female, Itsukaichi, Kariyose, 20-IV-1968, Y. Hasegawa (SM). Mt. Mitousan: 1 female, 4-V-1968, H. Takizawa; 1 male and 1 female, 27-V-1968, H. Takizawa (NIAES). 2 males and 2 females, Mt. Jinbasan, 6-V-1974, S. Miyakawa (SM). 1 male, Hinohara, Yazawa-rindou, 18-V-1997, H. Yoshitake (NIAES). Hachiouji: 3 males and 1 female, 1-V-1938, S. Miyakawa (SM); 1 male and 1 female, 1-V-1966 (SM); 2 males, 1-V-1966, S. Miyakawa (SM). Hachiouji, Uratakao, Kogesawa-rindou: 1 male, 30-IV-1938, S. Miyakawa (SM); 2 males and 2 females, 350–550 m, 35°38'30"–35°39'27" N, 139°11'57"–139°14'30" E, 17-V-2005, H. Yoshitake, on *R. crataegifolius* (NIAES). Okutama: 1 female, 9-V-1968, Hasegawa (SM); 1 male, 29-IV-1980, Y. Shiozaki (YS). 3 males and 4 females, Okutama, Kurasawadani, 6-V-1948, S. Miyakawa (SM). 2 males and 5 females, Okutama, Nippara, 24-V-1970, K. Unno (SM). 1 male and 1 female, Okutama, Unazawa, 7-V-1982, K. Shiozaki (YS). 2 males and 1 female, Okutama, Asamaone, 2-V-1992, K. Matsumoto (NIAES). KANAGAWA. 1 male, Yokohama, Motomachi, 29-VI-1970, S. Miyakawa (SM). 2 females, Hakone, Mt. Daigatake, Kozuka, 24-V-1979, S. Miyakawa (SM). Hadano, Mizunashigawa: 1 male, 29-IV-1983, K. Shiozaki (YS); 1 male, 29-IV-1983, Y. Shiozaki (YS). 1 female, Hadano, Yabitsu-touge Pass, 30-V-1983, K. Shiozaki (YS). 1 male, Yamakita, Mikuni-touge Pass, 23-V-1994, Y. Notsu (NIAES). 1 female, Tsukui, Kaminokawa-rindou, 21-V-1995, Y. Goto (NIAES). NIIGATA. 1 male, Kamikawa, 3-VI-2000, K. Takahashi (NIAES). FUKUI. 2 males and 5 females, Kanazu, Mt. Kariyasuyama, 20-V-1989, S. Inoue (ELKU). YAMAMASHI. 1 female, Mt. Mishotaisan, 17-VII-1940, S. Miyakawa (SM). 1 male and 1 female, Shirosu, 7-VI-1958 (ELKU). 1 female, Uenohara, 17-IV-1968 (SM). 1 male, Kawamura, Akiyama, 4-V-1969, S. Miyakawa (SM). 1 male, Lake Saiko, 27-V-1970, K. Unno (SM). 1 female, Ootsuki, Tomioka, 17-V-1976, S. Miyakawa (SM). 14 males and 19 females, Ootsuki, Koganezawa-rindou, 30-V-1980, J. Okuma (SM). 1 female, Mt. Fujisan, Subaru-line, 2000 m, 3-VI-1977, S. Miyakawa (SM). 1 female, Mt. Yatsugatake, 26-V-1979, A. Seki (NIAES). 16 males and 14 females, Koganezawa, 2-VI-1980, S. Miyakawa (SM). 1 female, Daibosatsu-touge Pass, 8-VI-1968, Y. Hasegawa (SM). Enzan, Hikawa-rindou: 1 male, 1-VII-1982, M. Sawai (SM); 1 male and 1 female, 2-VII-1982, M. Sawai (SM). 1 female, Mt. Kushigatayama, Maruyama-rindou, 23-IV-1984, S. Tsuyuki (NIAES). 2 males, Akeno, Manjuu-touge Pass, 6-VI-1986, S. Miyakawa (SM). 1 male, Kanayama, 1-VII-1989, T. Nonaka (NIAES). Ashiyasu, Hirogawara: 1 male, 24-V-1969, Y. Hasegawa (SM); 1 female, 8-VI-1989, H. Kojima (NIAES); 1 male and 1 female, 1529 m, 17-VI-1990, S. Miyakawa (SM); 1 female, 20-VI-1993, T. Horiguchi (NIAES). 1 male and 1 female, Nirasaki, Mt. Kayagatake, 16-V-1996, S. Miyakawa (SM). 1 female, Tabayama, Sanjounoyu, 20-V-1999 (NIAES). NAGANO. 1 female, Mt. Nyuukasayama, 6-VI-1942, S. Miyakawa (SM). 1 male and 3 females, Nagano, Uematsu, VI-1957, K. Matsuo (SM). 1 male and 1 female, Ina, Onasawa, 6-VI-1962, K. Oshima (ELKU). 1 male, Shimajimadani, VI-1970, N. Nino (SM). 1 female, Tobira-onsen, 26-VI-1974, H. Hayakawa (SM). 1 female, Kisofukushima, Higashiyama, 19-VII-1986, K. Matsui (SM). 1 female, Kisofukushima, 21-VIII-1988, K. Matsui (SM). 1 female, Kisofukushima, Uyama, 29-VIII-1988, K. Matsui (SM). 1 male, Kisofukushima, Kibio, 18-IX-1988, K. Matsui (SM). 1 male and 1 female, Kisokoma-kougen, Kibio, 21-VIII-1989, K. Matsui (SM). 1 male and 2 females, Nakakawa, 1-V-1998, H. Yoshitake (NIAES). 1 female, Hara, Pension Village, 2–4-VII-1998, H. Yoshitake (NIAES). GIFU. 1 male, Mt. Hakusan, 29-VI-2002, H. Hirano (NIAES). 1 female, Takane, Hiwada-kougen, 1-X-1989, K. Matsui (SM). 6 exs., Toki, 12-V-2010, K. Izawa, on *R. trifidus* (KI). SHIZUOKA. 1 female, Shinfuji, 30-IV-1989, N. Niwa (NIAES). 1 female, Honkawane, Sessokyou, 9–10-V-1992, T. Kishimoto (NIAES). Izu-hantou Peninsula: 2 males and 1 female, Kyuu-amagi-touge Pass, 3-V-1972, S. Miyakawa (SM); 2 males and 3 females, Mt. Nekkodake, 19-V-1974, S. Miyakawa (SM); 21 males and 16 females, Okuhara-rindou, 22-VI-1977, J. Okuma (SM); 2 females, Mt. Toogasayama, 17-V-1980, J. Okuma (SM); 1 male, Izu Okawa, 3-VI-1989, H. Takizawa (NIAES). AICHI. 8 males and 6 females, Kasugai, Hosono, 1-V-1991, H. Kojima (ELKU). Toyota, on *R. trifidus*: 5 exs., Oobora, Nikake-rindou, 3-V-2010, K. Izawa (KI); 6 exs., Nishiichinonochou, 8-V-2013, K. Izawa (KI); 7 exs., Asugawachou, Asugawa-hoan-rindou, 8-V-2013, K. Izawa (KI). MIE. Misugi, Hirakura: 1 male, 2-VI-1985, T. Imamura (NIAES); 3 females, 600–700 m, 15–17-V-2005, H. Hirano, on *R. crataegifolius* (NIAES). 1 male, Fujigawa, Mt. Suzugadake, 600 m, 3-V-2002, S. Arai (NIAES). 4 exs., Komonochou, Komono, 15-V-2013, K. Izawa, on *R. microphyllus* (KI). SHIGA. 1 male and 1 female, Mt. Ibukiyama, 220 m, 3-VI-1990, S. Miyakawa (SM). KYOTO. 1 male, Sugi-touge Pass, 29-V-1977, M. Sawai (SM). HYOGO. 1 male, Haga, Akasai-keikoku, 3-V-1993, S. Tsuyuki (NIAES). NARA. 1 female, Gose, Mt. Kongousan, 1100 m, 4-VII-1998, T. Kishimoto (NIAES). WAKAYAMA. 1 male and 1 female, Mt. Gomadanzan, 7–8-VI-1997, T. Ito (NIAES). 1 female, Nakahechi, Mizukami, 28-IV-2003, H. Hirano (NIAES). 1 male, Arida, Kamiyukawa, 19-V-1973, I. Matoba (ELKU). TOTTORI. 1 male and 1 female, Wakasa, Mt. Hyounosen, 7-VI-1987, K. Yoshihara (SM). SHIMANE. 1 female, Okinoshima I., Mt. Takuhiyama, 13-V-1975, J. Okuma (SM). OKAYAMA. 4 males and 1 female, Yubara, Yubara-onsen, 21-V-1991, H. Nakamura (NIAES). 1 male, Hokubou, Kamimizuta, 8-V-1982, K. Yoshihara (SM). 1 female, Iwayadani, 4-V-1981, K. Yoshihara (SM). 1 male and 1 female, Kawakami, Myouren-keikoku, 27-VI-1982, K. Yoshihara. HIROSHIMA. 1 female, Kouzan, Bessako, 10-V-1996 (ELKU). 1 female, Yoshiwa, 11-V-1976, K. Baba (ELKU). **Shikoku.** TOKUSHIMA. 1 male, Sanuki Mts., Mt. Ryuuousan, 17-VIII-1989, K. Matsumoto (NIAES). EHIME. 5 males and 5 females, Omogokei, 18–19-V-1968, K. Hatta (EUMJ). 1 male, Iyomishima, Tomisato, 3–5-VIII-1974, G. Tokihiro (EUMJ). 4 males and 6 females, Houjou, Kukawa, 22-IV-1977, A. Oda (EUMJ). **Kyushu.** FUKUOKA. Mt. Hikosan: 1 male, 18-VI-1965, K. Takeno (ELKU); 1 female, 12-VI-1965, S. Kimoto (ELKU); 1 female, 17-V-1971, M. T. Chujo (ELKU); 2 males and 1 female, 30-V-1995, K. Morimoto (ELKU). 1 male, Mt. Tachibanayama, 23-IV-1997, H. Kojima (ELKU). SAGA. 1 female, Sefuri Mts., 28-IV-2002, H. Hirano (NIAES). 16 males and 13 females, Mt. Kusenbuyama, Hakotani-rindou, 8-V-2005, H. Yoshitake, on *R. crataegifolius* (NIAES). NAGASAKI. Tsushima I.: 1 female, Mt. Ooboshiyama, 16-VII-1981, Y. Syouno (ELKU); 1 male, Izuhara, Mt. Ariakeyama, 5–9-V-1997, H. Yoshitake (NIAES). 2 males and 1 female, Tashirobaru, 29-IV-1979, S. Imasaka (NIAES). 1 male, Taradake Mts., Mt. Gokaharadake, 12-VI-1979, S. Imasaka (NIAES). Shimabara, Sanbuki: 1 male and 1 female, 7-V-1976, S. Imasaka (NIAES); 1 male, 10-V-1976, S. Imasaka (NIAES); 6 females, 17-V-1976, S. Imasaka (NIAES); 1 female, 21-V-1976, S. Imasaka (NIAES); 1 male and 2 females, 22-V-1976, S. Imasaka (NIAES). Shimabara, Akamatsudani: 3 females, 24-V-1980, S. Imasaka (NIAES); 1 male, 27-V-1980, S. Imasaka (NIAES). 2 females, Sasebo, Mt. Hattendake, 23-V-1982, S. Miyakawa (SM). 3 males and 2 females, Kawatana, Kobagou, 27-V-1984, J. Okuma (SM). OITA. Kuju Mts., Mt. Kurodake: 1 female, 29-VII-1979, S. Imasaka (NIAES); 1 male, 9-VI-1985, K. Konishi (ELKU); 2 males, Oike-enchi, 7-VI-2002, H. Hirano (NIAES). 1 female, Kuju Mts., Jizoubaru, 28-V-1987, K. Morimoto (ELKU). 1 male, Kuju Mts., Bougatsuru, 27-V-1988, K. Morimoto (ELKU). 1 male and 2 females, Kuju Mts., Choujabaru, 22-V-1999, K. Morimoto (ELKU). MIYAZAKI. 1 male, Ebino, Shiratori-onsen, 30-IV-1991, K. Morimoto (ELKU).

#### Distribution.

China (Liaoning and Heilongjiang – new record), Korea (North – new record, Central, South, and Ulreungdo Island), Japan (Hokkaido, Honshu, Izu Islands, Shikoku, Kyushu, and Tsushima Island) (Fig. 133).

#### Biological note.

Adults of this common species were collected from *Rubus idaeus* L. subsp. *melanolasius* Focke f. *concolor* (Kom.) Ohwi in Korea (Korotyaev and Hong 2004). In addition, we collected a number of adults at several localities in the southern Korean Peninsula, mainly from *Rubus crataegifolius* Bunge with flower buds and *Rubus pungens* Camb. var. *oldhamii* (Miq.) Maxim. with flowers. In Japan, adults have been collected on many occasions from *Rubus* species, such as *Rubus crataegifolius*, *Rubus microphyllus*, and *Rubus trifidus*. In Toyosawa-rindou, Iwate, Honshu (Fig. 135), a number of adults (Fig. 138) were found on *Rubus crataegifolius* (Fig. 136) and observed feeding on the leaves and flowers of the plant (Fig. 137).

### Scleropteroides longiprocessus

Taxon classificationAnimaliaColeopteraCurculionidae

Huang & Yoshitake sp. n.

http://zoobank.org/C8AC65A6-71E7-4CD0-9387-CFA00B598B09

Figs 3 , 4 , 26 –29 , 36 –37 , 70 –89 , 134

#### Diagnosis.

**Figures 70–79.**
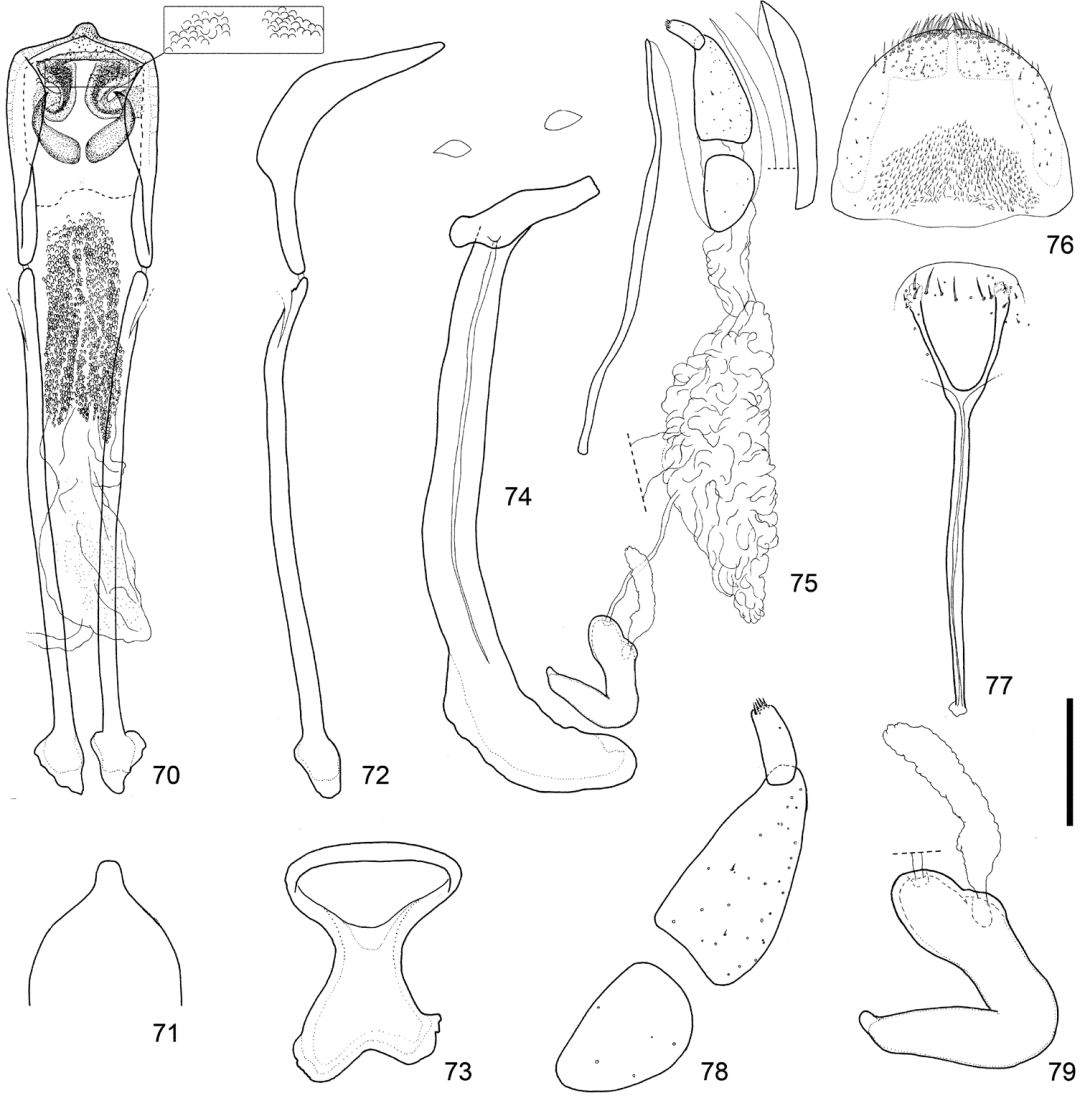
Male and female terminalia of *Scleropteroides longiprocessus* Huang & Yoshitake, sp. n. from Mt. Kusenbuyama, Kyushu, Japan. **70** Aedeagus, dorsal view **71** Apex of the penis, dorsal view **72** Aedeagus, lateral view **73** Tegmen **74** Sternites VIII and IX, male **75** Female terminalia and genitalia, lateral view **76** Tergite VIII, female **77** Sternite VIII, female **78** Coxite and stylus **79** Spermatheca. Scale: 0.20 mm for **70–77**, 0.10 mm for **78–79.**

**Figures 80–89.**
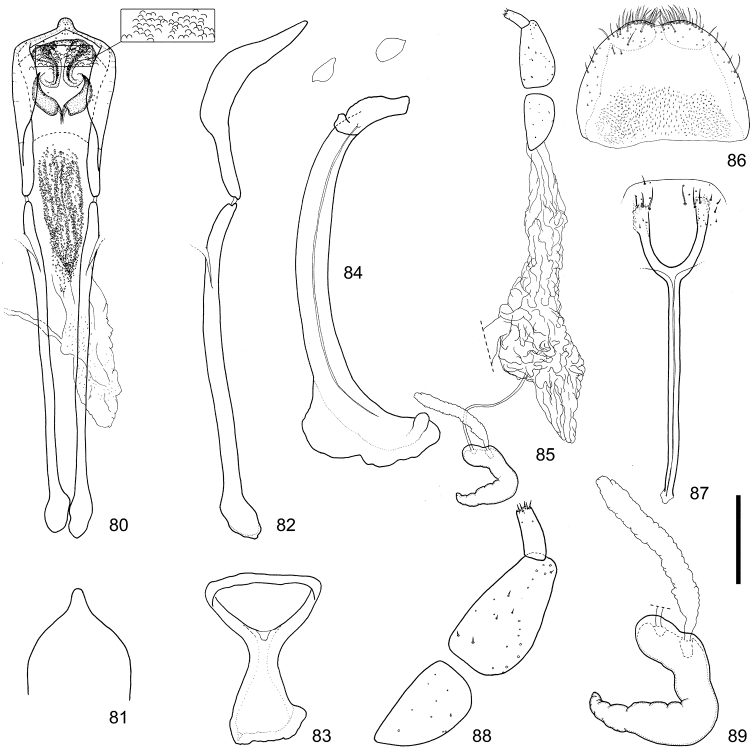
Male and female terminalia of *Scleropteroides longiprocessus* Huang & Yoshitake, sp. n. from Tochigi, Honshu, Japan. **80** Aedeagus, dorsal view **81** Apex of the penis, dorsal view **82** Aedeagus, lateral view **83** Tegmen **84** Sternites VIII and IX, male **85** Female genitalia, lateral view **86** Tergite VIII, female **87** Sternite VIII, female **88** Coxite and stylus **89** Spermatheca. Scale: 0.20 mm for **80–87**, 0.10 mm for **88–89.**

The species is very similar to *Scleropteroides hypocrita* but is distinguished from it by the following characteristics: subapical part of the prothorax strongly constricted (Fig. 36); elytra with strongly prominent humeri, straightly convergent toward the subapical calli, (Fig. 37); scales in a row on elytral intervals semierect, nearly as long as the width of each interval (Fig. 37); apical half of the male rostrum slightly more widened (Fig. 26); male metaventrite more prominent ventrally along the apico-lateral margin of the metaventral receptacle; male ventrite I with no prominence; penis with a longer apical projection (Figs 71, 81); endophallus with a pair of narrow falcate sclerites in the basal part (Figs 70, 80); female sternite VIII with arms that are divergent apically (Figs 77, 87); bursa copulatrix without minute coniform spicules in the posterior part (Figs 75, 85).

#### Male.

LB: 2.27–2.63 (mean, 2.39). LR: 0.97–1.08 (mean, 1.04). WP: 0.95–1.00 (mean, 0.97). LP: 0.76–0.85 (mean, 0.82). WE: 1.49–1.62 (mean, 1.57). LE: 1.53–1.66 (mean, 1.62). N = 10 for all measurements. Habitus as shown in Figs 3–4.

*Vestiture*. Clavate scales slightly longer and semierect on head, basal 2/3 of rostrum, and pronotum. Scales on elytral intervals (Fig. 37) semierect, slightly longer, 0.9–1.2 × as long as interval width.

*Rostrum* (Figs 26–27) slender, 1.21–1.32 × as long as pronotum; sides more strongly widened in apical part, which is 1.23 × as wide as basal part. Antennae (Fig. 27) with length ratio of funicular segments I: II: III: IV: V: VI = 1.88: 1.54: 1.12: 1.00: 0.96: 0.96 and width ratio = 1.73: 1.00: 1.36: 1.45: 1.45: 1.55.

*Pronotum* (Fig. 36) 1.13–1.27 × as wide as long, 0.47–0.52 × as long as and 0.59–0.65 × as wide as elytra; subapical constriction stronger; sides subparallel along basal half, then rapidly convergent toward apex.

*Elytra* 1.01–1.04 × as wide as wide, 1.92–2.14 × as long as and 1.54–1.69 × as wide as pronotum, straightly convergent toward subapical calli; humeral calli slightly more prominent; subapical calli moderately prominent.

*Underside*. Metaventrite moderately prominent ventrally along apico-lateral margin of metaventral receptacle. Ventrite I weakly flattened on disc, lacking prominence; length ratio of ventrites I: II: III: IV: V = 4.33: 2.00: 1.00: 1.13: 2.22 and width ratio = 1.89: 1.70: 1.23: 1.11: 1.00.

*Genitalia*. Tegmen (Figs 73, 83) with apodeme strongly widened toward apex. Penis (Figs 70–72, 80–82) thick in profile; sides more or less weakly widened from base to apical 1/5, strongly convergent apically; apical projection more slender. Endophallus (Figs 70, 80) with pair of narrow falcate sclerites in basal part.

Otherwise as in *Scleropteroides hypocrita*.

#### Female.

LB: 2.08–2.53 (mean, 2.34). LR: 0.97–1.15 (mean, 1.09). WP: 0.90–1.07 (mean, 0.97). LP: 0.74–0.86 (mean, 0.81). WE: 1.43–1.64 (mean, 1.54). LE: 1.46–1.68 (mean, 1.59). N = 10 for all measurements.

*Rostrum* (Figs 28–29) slightly more slender, 1.30–1.39 × as long as pronotum.

*Pronotum* 1.14–1.28 × as wide as long.

*Elytra* 1.01–1.08 × as long as wide.

*Underside*. Ventrite V with small median concavity, which is sometimes obscure. Sternite VIII (Figs 77, 87) with arms slightly more slender and apically divergent.

*Genitalia*. Bursa copulatrix (Figs 75, 85) lacking spicules. Coxites (Figs 78, 88) nearly 5.0 × as long as styli; styli more slender, nearly three times as long as wide. Spermatheca (Figs 79, 89) with collum more strongly convex; insertions of duct and gland slightly more distant from each other.

Otherwise showing almost the same sexual dimorphism as in *Scleropteroides hypocrita*.

#### Type material.

HOLOTYPE: 1 male (NIAES), “[Japan: Kyushu]/Mts. Sefuri-sanchi/Mt. Kusenbu-yama/8-V-2005/Hiraku Yoshitake” (printed on white card); “On *Rubus palmatus* Thunb. (Rosaceae)/[JN: Nagaba-momiji-ichigo] with flowers” (print ed on white card); “[ HOLOTYPE ] Male/*Scleropteroides*/*longiprocessus*/Huang & Yoshitake, 2008” (typed on red card). PARATYPES. **JAPAN: Honshu.** IWATE. 1 male and 1 female, Miyako, Genbehdaira, 9-VI-2007, K. Morimoto (NIAES). 2 males and 6 females, Tsuchisaka-touge Pass, Kawai, 12-VI-2007, J. Kantoh (NIAES). MIYAGI. 1 male, Naruse, Miyatojima I., V-1995, M. Kodama (NIAES). 1 male and 2 females, Sendai, Mt. Banzan, 20-V-1995, H. Yoshitake (NIAES). AKITA. 1 male and 1 female, Lake Tazawako, 15-VI-1974, S. Miyakawa (SM). YAMAGATA. 2 males and 3 females, Mt. Zaousan, Yoshikari-rindou, 18-VI-1983, S. Miyakawa (SM). FUKUSHIMA. Haranomachi, Kozikiishi-rindou: 1 male, 30-V-1987, H. Ebihara (NIAES); 1 male, 5-VI-1988, S. Miyakawa (SM). 1 female, Haranomachi, Akanesawa-rindou, 5-VI-1988, S. Miyakawa (SM). 1 female, Namie, 30-IV-1994, H. Yoshitake (NIAES). 1 male, Iizaka, Moniwa, Yakematsu, 1-V-1997, S. Saito (NIAES). IBARAKI. Mt. Yamizosan, Shimokitazawa: 7 males and 1 female, 1022 m, 28-V-1988, S. Miyakawa (SM); 1 male and 2 females, 28-V-1988, Y. Kurosawa (SM). TOCHIGI. 1 male and 2 females, Nikko, Kashiwagi, 16-V-1982, S. Miyakawa (SM). 1 male and 1 female, Shiobara, Hikinuma, 19-V-1989, S. Miyakawa (SM). 3 males and 1 female, Mt. Shibakusayama, 18-V-1990, H. Kojima (ELKU). GUNMA. 1 male and 1 female, Matsuida, Kirizumi-onsen, 16-V-1998, S. Arai (NIAES). 2 males and 2 females, Niiharu, Amemi-rindou, 4-V-1998, S. Arai (NIAES). 1 female, Fujiwara, Nakanosawa-rindou, 19-V-1989, S. Miyakawa (SM). 6 males and 4 females, Fujiwara, Midorizawa-rindou, 29-V-1989, S. Miyakawa (SM). NIIGATA. Kurokawa: 1 female, 28-IV-1973, K. Baba (ELKU); 2 females, 26-V-1976, K. Baba (ELKU); 1 female, 24-V-1980, K. Baba (ELKU). 1 male and 1 female, Teradomari, Enjouji, 21–23-V-1996, K. Ishida (NIAES). 1 male, Sadogashima I., Nagaishihama, 22-VII-1970, K. Baba (ELKU). FUKUI. 2 males and 1 female, Obama, Shimonegouri, 6-V-1979, H. Sasaji (ELKU). YAMANASHI. 1 female, Mt. Fujisan, Fuji-rindou, 23-VI-1983, S. Miyakawa (SM). 1 male, Ootsuki, Nanaho, 9-V-1976, S. Miyakawa (SM). 1 female, Ootsuki, Koganezawa-rindou, 30-V-1980, J. Okuma (SM). NAGANO. 1 female, Tobira-onsen, 26-VI-1985, H. Hayakawa (SM). 5 males, Nagiso, 29-IV-1998, H. Yoshitake (NIAES). 2 males and 1 female, Iida, Hatouchi-rindou, 29–30-IV-1998, H. Yoshitake (NIAES). 2 males, Nakakawa, 1-V-1998, H. Yoshitake (NIAES). GIFU. Nakatsugawa, Kamisaka: 1 female, 13-VI-1987, S. Miyakawa (SM); 1 female, 13-VI-1987, K. Morimoto (ELKU). 5 exs., Nakatsugawa, Nenoue-kougen, 6-V-2013, K. Izawa, on *R. palmatus* var. *coptophyllus* (KI). SHIZUOKA. 1 female, Shinfuji, 30-IV-1989, N. Niwa (NIAES). 5 males and 6 females, Nakaizu, Mt. Amagisan, 3-V-1990, H. Kojima (ELKU). 1 male and 4 females, Mt. Ooyama, 5-V-1993, Y. Notsu (NIAES). 3 males and 1 female, Shizuoka, Umegashima, 15-V-1993, T. Kishimoto (NIAES). Izu-hantou Peninsula: 3 males and 1 female, Hacchouike Pond, Amagi-touge Pass, 28-V-1972, S. Miyakawa (SM); 1 female, Kamo, Oogusu, 11-V-1980, J. Okuma (SM); 1 female, Hacchouike Pond–Kantenbashi, 14-V-1980, J. Okuma (SM). 1 male, Umegashima, 15–16-V-1993, H. Sato (NIAES). 1 female, Shizuoka, Nakaizu, Amagi-kougen, 31-V-1987, S. Miyakawa (SM). AICHI. 6 exs., Shitara, Mt. Nishinagura-Iyama, 29-V-2010, K. Izawa, on *R. palmatus* var. *coptophyllus* (KI). Toyota, on *R. palmatus* var. *coptophyllus*: 5 exs., Nishiichinonochou, 8-V-2013, K. Izawa, (KI); 4 exs., Asugawachou, Asugawa-hoan-rindou, 8-V-2013, K. Izawa (KI); 2 exs., Inabuchou, Noirigawa, 22-V-2013, K. Izawa (KI). SAITAMA. 1 female, Mt. Kasayama, 26-V-1967, H. Takizawa (NIAES). 1 male, Saitama Yokote, 28-IV-1974, S. Miyakawa (SM). 1 female, Lake Miyazawako, 11-V-1975, S. Miyakawa (SM). 1 male, Shoumaru-touge Pass, 9-V-1976, S. Miyakawa (SM). CHIBA. 1 female, Mt. Kiyosumiyama–Kenminnomori, 31-V-1979, J. Okuma (SM). TOKYO. 1 female, Okutama, Kurasawadani, 19-VI-1938, S. Miyakawa (SM). Nishitama, Mt. Oodakeyama: 1 male, 21-V-1942, S. Miyakawa (SM); 2 males and 1 female, 15-V-1966, S. Miyakawa (SM). Hachiouji, Mt. Takaosan: 1 female, 30-V-1980, Y. Shiozaki (YS); 1 male and 1 female, 23-VI-1996, H. Yoshitake (NIAES); 5 exs., 22-V-2010, K. Izawa, on *R. palmatus* var. *coptophyllus* (KI). Hachiouji, Mt. Takaosan, Kogesawa-rindou: 3 males, 14-V-1944, S. Miyakawa (SM); 1 male and 1 female, 1–2-V-1999, H. Yoshitake (NIAES). 5 males and 5 females, Hachiouji, Mt. Takaosan, Path 6, 240–599 m, 35°37'30"–35°37'50"N, 139°14'36"–139°15'43"E, 15-V-2005, *R. palmatus* var. *coptophyllus*, H. Yoshitake (NIAES). 1 female, Hachiouji, Kitano, 14-IV-1968, S. Miyakawa (SM). 1 female, Inagi, Yomiuri Land, 17-V-1974, S. Miyakawa (SM). KANAGAWA. 1 male, Mikuni-touge Pass, 7-VI-1970, H. Takizawa (NIAES). 1 female, Mt. Ooyama, 2-VI-1981, S. Miyakawa (SM). 2 males, Yokohama, Nakayama, 3-V-1983, S. Miyakawa (SM). 1 female, Hakone, Oowakudani, 15-VI-1985, S. Tsuyuki (NIAES). Hadano: 1 female, Mt. Koubouyama, 24-IV-1983, Y. Shiozaki (YS); 1 male, 30-IV-1990, H. Takizawa (NIAES). 1 female, Tanzawa Mts., Mizunashigawa, 30-IV-1983, Y. Shiozaki (YS). Hadano, Yabitsu-touge Pass: 1 female, Yabitsu, 1-VII-1983, K. Shiozaki (YS); 1 female, 29-IV, K. Suzuki (ELKU). YAMANASHI. 1 female, Akaishi-sanmyaku Range, Norogawa-rindou, 5-VI-1983, Y. Shiozaki (YS). MIE. 1 female, Ninomata–Owase, 26-VI-1960, H. Ichihashi (NIAES). 1 male, Komono, Unbomine, 28-IV-1985, A. Amagasu (ELKU). 1 female, Shiga, Mt. Ibukiyama, 10-VI-1990, H. Takizawa (NIAES). 1 female, Toba, Mastuno, Mt. Aonomineyama, 19-V-1996, H. Yoshitake (NIAES). 8 males and 6 females, Toba, Toushijima I., 10-IV-1996, K. Ishida (NIAES). 1 male, Misugi, Hirakura, 7–8-VI-1997, H. Yoshitake (NIAES). NARA. 1 female, Kawakami, Mt. Wasamatayama, 8-VII-1995, A. Yoshida (NIAES). 1 male, Mt. Miyama, 7-VII-1989, S. Yamada (NIAES). OKAYAMA. 2 males and 1 female, Kamo, Kurami, 29-VI-1985, K. Watanabe (SM). 2 females, Tetta, Mt. Aratoyama, 16-V-1982, K. Yoshihara (SM). 1 male, Takahashi, Mt. Gagyuusan, 5-V-1984, K. Yoshihara (SM). **Izu Islands.** 1 female, Kouzushima I., Maehama–Tsuzukizawa, 9-V-1979, J. Okuma (SM). **Shikoku.** TOKUSHIMA. 1 female, Haranomachi, 8-VI-1980, K. Shiozaki (YS). 1 male and 1 female, 1-VI-1986, A. Watanabe (YS); 1 female, 5-VI-1986, A. Watanabe (YS). EHIME. 2 females, Imabaru, Mt. Takanawazan, 5-V-1976, S. Inoue (EUMJ). KOCHI. 1 male, Okunanokawa, 4-V-1967, H. Oonishi (EUMJ). Cape Murotomisaki: 1 male and 1 female, 7-VI-1959, M. Miyatake (EUMJ); 1 female, 8-V-1959, S. Hisamatsu (EUMJ); 2 females, 12-VII-1961, M. Miyatake (NIAES). **Kyushu.** FUKUOKA. 1 male, Chikugo, Mt. Kumadoyama, 1-VI-1958, Y. Miyaue (ELKU). 1 female, Munakata, Mt. Jouyama, 6-IV-1975, K. Kido (ELKU). 1 female, Itoshima, Himeshima I., 3-VI-1990, K. Kido (ELKU). 1 female, Shikanoshima I., 29-IV-1994, K. Morimoto (ELKU). 1 female, Fukuoka, Motooka, 2-VII-1994, K. Morimoto (ELKU). 1 male and 1 female, Mt. Sefurisan, 3-VI-2001, H. Hirano (NIAES). 1 male, Yabe, Mt. Shakagatake, 25-IV-2004, H. Hirano (NIAES). 3 males and 1 female, Nokonoshima I., 17-VI-2003, H. Hirano (NIAES). Mt. Hikosan: 2 males and 3 females, 17–19-V-1967, H. Takizawa (NIAES); 1 female, 4-V-1985, Y. Kajida (NIAES); 2 males and 4 females, 30-V-1995, K. Morimoto (ELKU); 1 female, 26-VI-2003, H. Hirano (NIAES). SAGA. 1 male, Okuhiratani, Mt. Taradake, 23-IV-1989, T. Yasunaga (ELKU). 2 males and 3 females, same data as holotype (NIAES). 2 males and 2 females, Mt. Kusenbuyama, Hakotani-rindou, 8-V-2005, H. Yoshitake, on *R. crataegifolius* (NIAES). NAGASAKI. 1 male, Shimabara, Taruki, 16-VII-1976, S. Imasaka (NIAES). 1 female, Shimabara, Akamatsudani Gorge, 8-VI-1979, S. Imasaka (NIAES). Mt. Unzendake: 1 female, 25-V-1976, S. Imasaka (NIAES); 1 female, 21-VII-1976, S. Imasaka (NIAES). 1 male and 2 females, Nomozeki, 13-V-1976, S. Imasaka (NIAES). 1 male, Tashirobaru, 21-IV-1977, S. Imasaka (NIAES). 15 males and 12 females, Sasebo, Mt. Eboshidake, 13-IV-1977, J. Okuma (SM). 4 males, Sasebo, Mt. Hattendake, 23-V-1982, J. Okuma (SM). Sasebo, Mt. Yahirodake: 1 male and 1 female, 18-IV-1977, J. Okuma (SM); 2 males, 6-IV-1981, J. Okuma (SM); 1 female, 21-V-1981, J. Okuma (SM); 1 female, 26-V-1982, J. Okuma (SM); 1 female, 29-III-1983, J. Okuma (SM); 1 female, 30-IV-1983, J. Okuma (SM); 1 male, 2-V-1983, J. Okuma (SM); 2 females, 15-V-1984, J. Okuma (SM). 1 male and 1 female, Hiradoshima I., Mt. Yasumandake, 10-V-1980, S. Imasaka (NIAES). KUMAMOTO. 1 male, Gokanoshou, 26–27-V-1978, K. Ohara (ELKU). Izumi, Mt. Shiratoriyama: 1 female, 1300 m, 6-VI-1980 (ELKU); 1 female, 7–8-VI-1989, T. Yasunaga (ELKU). 1 male and 2 females, Yabe, Naidaijin-rindou, 21-VI-1998, H. Kojima (ELKU). OITA. 1 male and 2 females, Kuju Mts., Bougatsuru, 27-V-1988, K. Morimoto (ELKU). KAGOSHIMA. Oosumi-hantou Peninsula, Cape Satamisaki: 1 male, 25-VI-1957, T. Saigusa (ELKU); 1 female, 8-VI-1958, H. Ueno (ELKU). 5 males and 6 females, Mt. Hoyoshidake, 14–15-IV-2002, H. Yoshitake (NIAES). 1 male, Mt. Kobadake, 28-VI-2003, H. Yoshitake (NIAES).

#### Distribution.

Japan (Honshu, Izu Islands, Shikoku, and Kyushu; Fig. 134).

#### Etymology.

The species name refers to the elongate apical projection of the penis, from Latin: *longi*-, from *longus*, -*a*, -*um*, meaning “long”, and *processus*, meaning “projection”. To be treated as a substantive in apposition.

#### Biological note.

In several localities in Honshu, a number of adults of this species were found on *Rubus palmatus* Thunb. var. *coptophyllus* (A.Gray) Kuntze ex Koidz. In addition, adults of this species were collected mostly from *Rubus palmatus* Thunb. and rarely from *Rubus crataegifolius* Bunge on Mt. Kusenbuyama, Kyushu.

### Scleropteroides horridulus

Taxon classificationAnimaliaColeopteraCurculionidae

(Voss, 1958)

Figs 5 , 6 , 30 –33 , 38 –39 , 90 [Fig F11] [Fig F12] –132 , 134

Homorosoma horridula Voss, 1958: 67 (type locality: China, “Kuatun”) (incorrect original spelling). – Chûjô 1971: 34 (Is. Okinawa: Nago).
Homorosoma horridulum (mandatory correction, gender agreement): Chûjô and Voss 1960: 7 (in part; Is. Okinawa: Nago). – Colonnelli 2004: 34 (in catalog; China, Japan).
Rhytidosomus insulare Voss 1971: 54 (type locality: Japan, Is. Amami, Hatsuno) (incorrect original spelling).
Scleropteroides horridulus : Korotyaev et al. 2014: 99 (combination from *Homorosoma*; Taiwan) (mandatory correction, gender agreement).
Scleropteroides insulare : Morimoto 1984: 316 (Amami, Okinawa); 1989: 514 (in checklist; Amami, Okinawa) (incorrect subsequent spelling).
Scleropteroides insularis : Yoshitake et al. 2004: 106 (in checklist).
Scleropteroides hypocrita : Colonnelli 2004: 34 (in part; not Hustache 1916).

#### Diagnosis.

**Figures 90–99.**
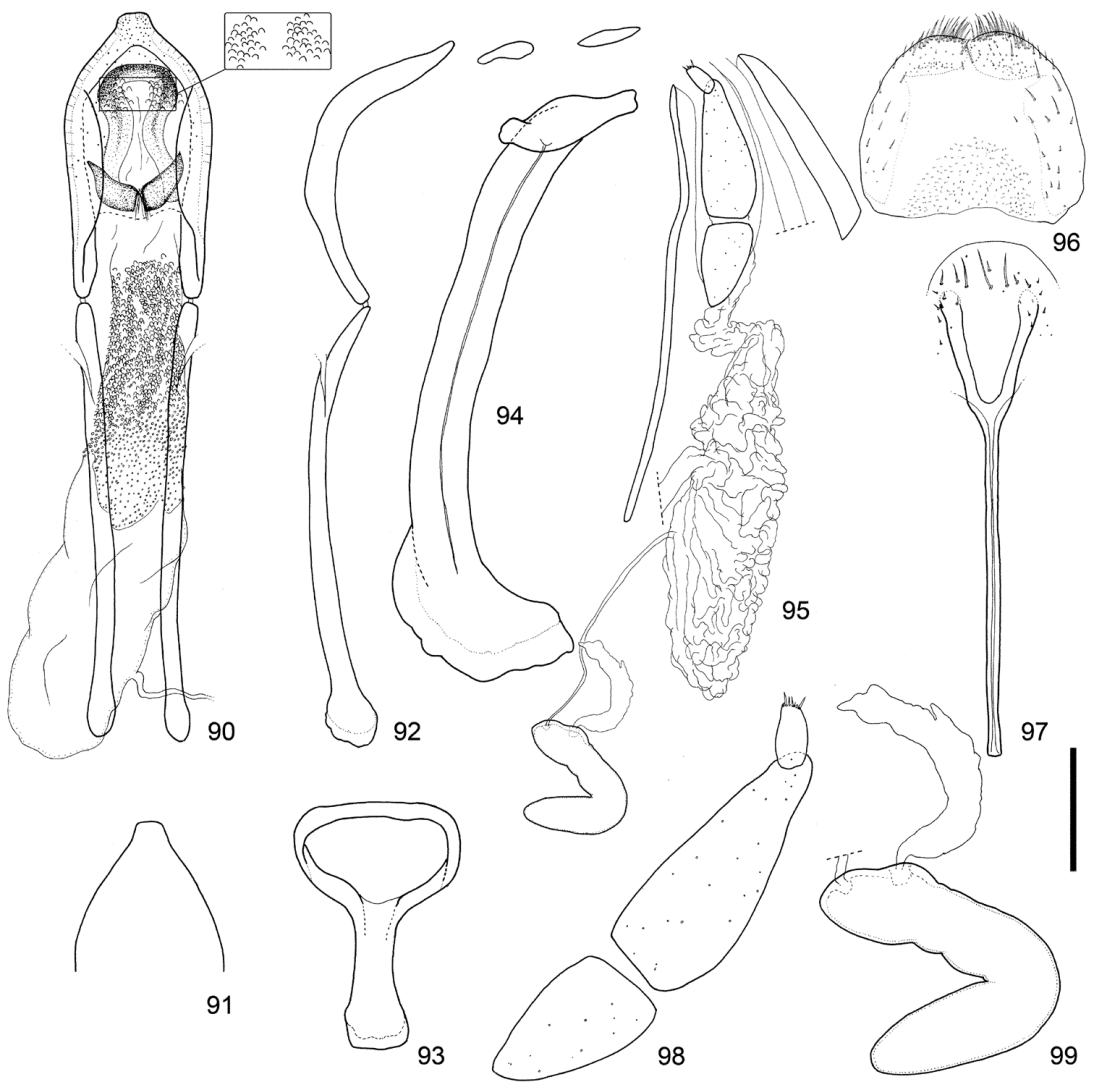
Male and female terminalia of *Scleropteroides horridulus* (Voss) from Amami-Oshima Island, the Ryukyus, Japan. **90** Aedeagus, dorsal view **91** Apex of the penis, dorsal view **92** Aedeagus, lateral view **93** Tegmen **94** Sternites VIII and IX, male **95** Female terminalia and genitalia, lateral view **96** Tergite VIII, female **97** Sternite VIII, female **98** Coxite and stylus **99** Spermatheca. Scale: 0.20 mm for **90–97**, 0.10 mm for **98–99.**

**Figures 100–109. F11:**
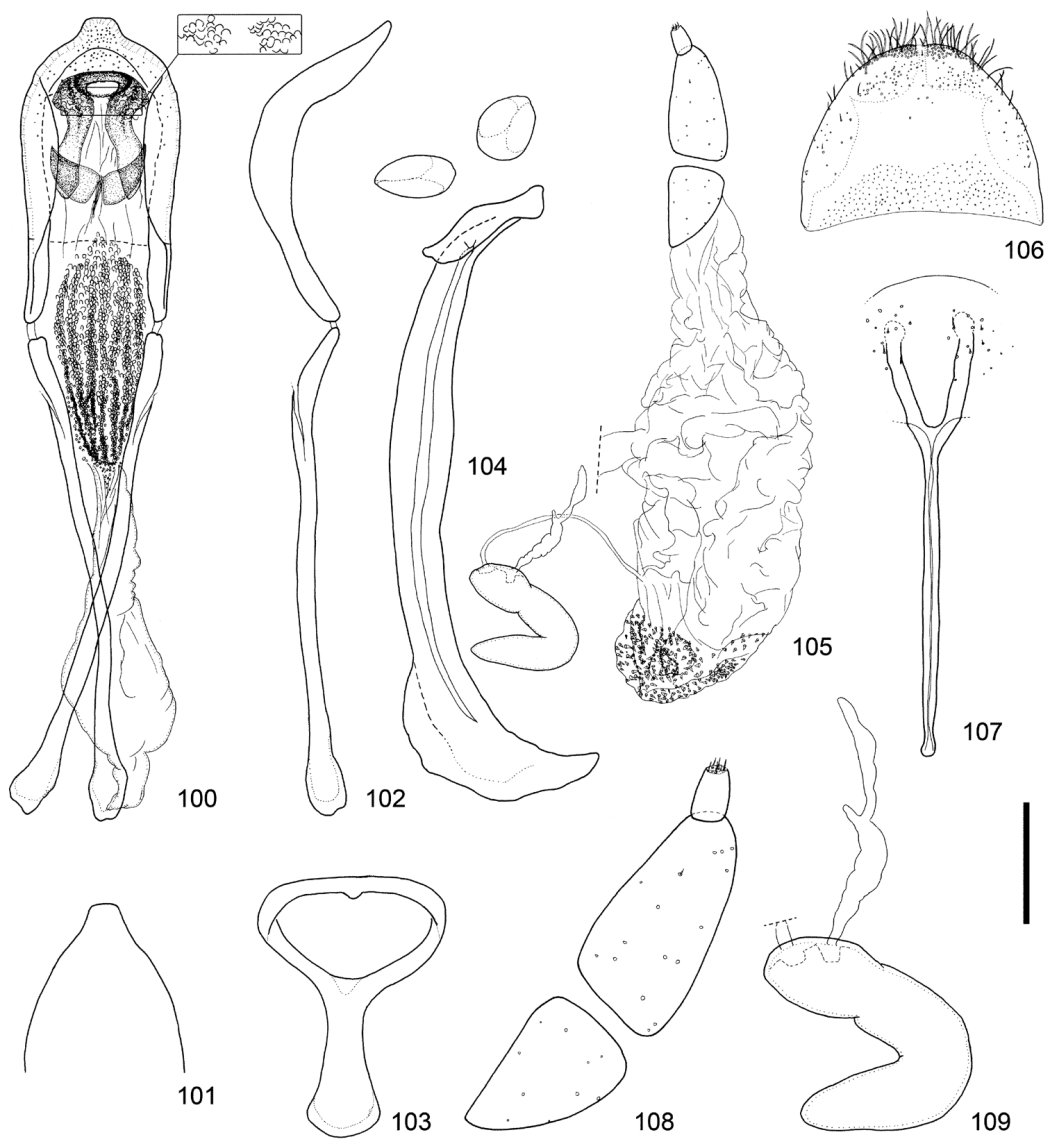
Male and female terminalia of *Scleropteroides horridulus* (Voss) from Fujian, southeastern China. **100** Aedeagus, dorsal view **101** Apex of the penis, dorsal view **102** Aedeagus, lateral view **103** Tegmen **104** Sternites VIII and IX, male **105** Female genitalia, lateral view **106** Tergite VIII, female **107** Sternite VIII, female **108** Coxite and stylus **109** Spermatheca. Scale: 0.20 mm for **100–107**, 0.10 mm for **108–109.**

This species differs from *Scleropteroides hypocrita* on the basis of the following characters: subapical part of the prothorax strongly constricted (Fig. 38); elytra with strongly prominent humeri, straightly convergent toward the subapical calli (Fig. 39); scales in a row on the elytral intervals erect, much longer than the width of each interval (Fig. 39); male metaventrite more strongly prominent ventrally along the apico-lateral margin of the metaventral receptacle; male ventrite I with no prominence; apical projection of the penis truncate at the apex (Figs 91, 101, 111, 114, 117, 120, and 123); basal part of the endophallus with a pair of weakly falcate sclerites (Figs 90, 100); female sternite VIII with apically divergent arms (Figs 97, 107); bursa copulatrix usually without minute coniform spicules (Figs 95, 105).

**Figures 110–124. F12:**
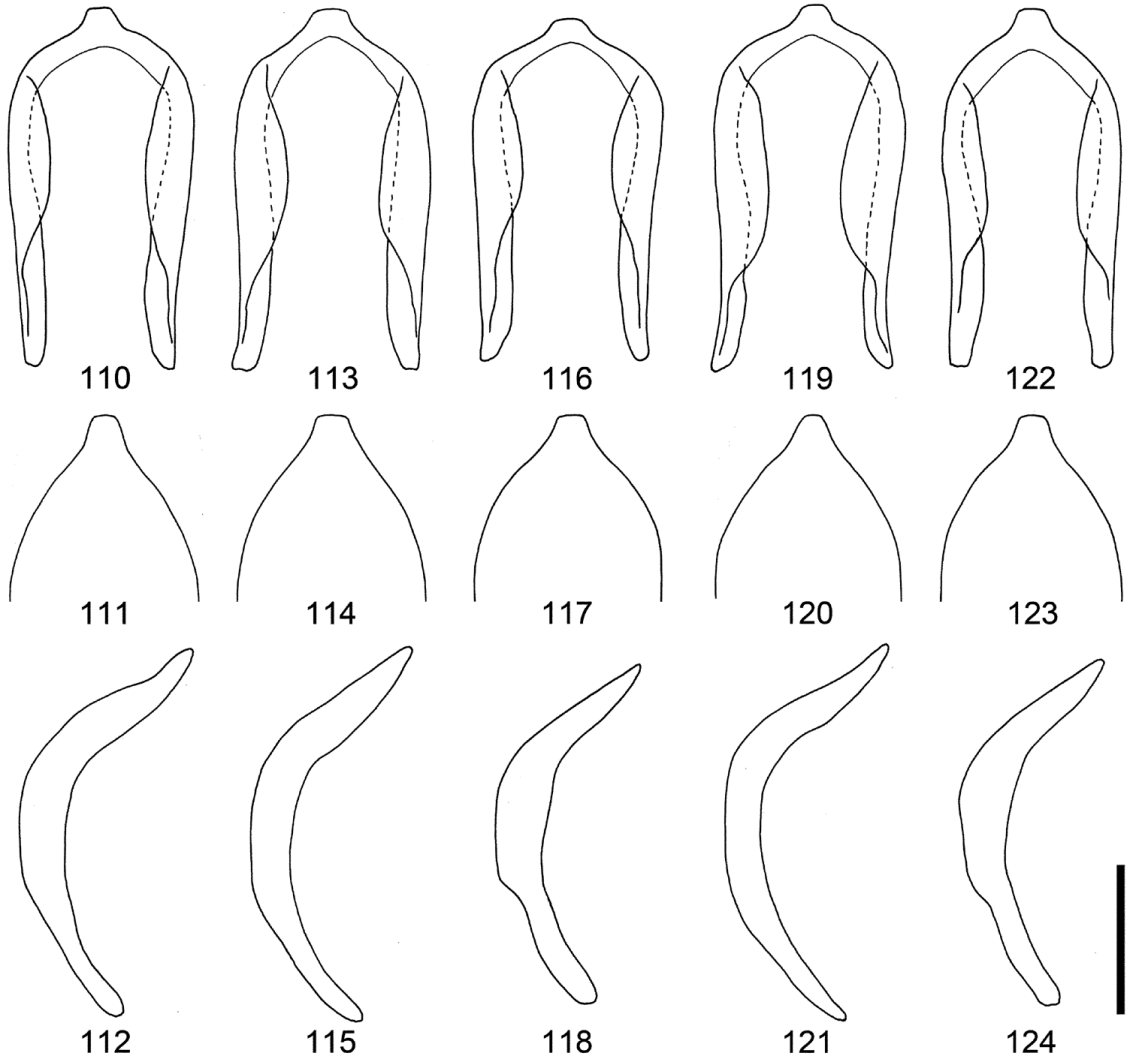
Male genitalia of *Scleropteroides horridulus* (Voss) from various localities. **110–112** Taiwan, China **113–115** Fujian, southeastern China **116–118** Hunan, central China **119–121** Yakushima Island, Nansei Islands, Japan **122–124** Mt. Jirisan, South Korea **110, 113, 116, 119, 122** Penis, dorsal view **111, 114, 117, 120, 123** Apex of the penis, dorsal view **112, 115, 118, 121, 124** Penis, lateral view. Scale: 0.20 mm.

#### Male.

LB: 2.40–2.71 (mean, 2.50). LR: 1.04–1.18 (mean, 1.12). WP: 0.94–1.10 (mean, 1.00). LP: 0.85–0.95 (mean, 0.90). WE: 1.61–1.91 (mean, 1.69). LE: 1.64–1.83 (mean, 1.69). N = 11 for all measurements. Habitus as shown in Figs 5–6.

*Vestiture*. Clavate scales long and erect on head, basal 2/3 of rostrum, and pronotum. Hair-like scales long and semierect on apical 1/3 of rostrum. Scales on elytral intervals (Fig. 39) erect, long, 1.3–1.5 × as long as interval width. Clavate scales on tibiae erect.

*Rostrum* (Figs 30–31) slender, 1.15–1.27 × as long as pronotum, sides slightly widened in apical part, which is 1.16× as wide as basal part. Antennae (Fig. 31) with length ratio of funicular segments I: II: III: IV: V: VI = 1.86: 1.50: 1.21: 1.00: 1.00: 0.93 and width ratio = 1.60: 1.00: 1.13: 1.20: 1.33: 1.47.

*Pronotum* (Fig. 38) 1.06–1.19 × as wide as long, 0.50–0.56 × as long as and 0.56–0.65 × as wide as elytra; subapical constriction strong; sides subparallel in basal 2/3, then strongly convergent toward apex.

*Elytra* 0.96–1.04 × as long as wide, 1.79–1.98× as long as and 1.54–1.79× as wide as pronotum, straightly convergent toward subapical calli; humeral calli strongly prominent; subapical calli moderately prominent.

*Underside*. Metaventrite moderately prominent ventrally along apico-lateral margin of metaventral receptacle. Venter coarsely and very densely punctured; ventrite I weakly flattened on disc, lacking prominence; ventrite V with more or less deeper median concavity; length ratio of ventrites I: II: III: IV: V = 3.56: 2.67: 1.00: 1.00: 2.56 and width ratio = 1.90: 1.76: 1.30: 1.20: 1.00.

*Genitalia*. Tegmen (Figs 93, 103) with apodeme slightly widened toward apex. Penis (Figs 90–92, 100–102, 110–124) with sides more or less weakly widened toward apical 1/5, then strongly convergent apically; apical projection truncate at apex. Basal part of endophallus (Figs 90, 100) with pair of weakly falcate sclerites.

Otherwise as in *Scleropteroides hypocrita*.

#### Female.

LB: 2.34–2.92 (mean, 2.68). LR: 1.06–1.36 (mean, 1.30). WP: 0.95–1.14 (mean, 1.09). LP: 0.88–1.04 (mean, 0.96). WE: 1.63–1.97 (mean, 1.82). LE: 1.62–2.03 (mean, 1.83). N = 16 for all measurements.

*Rostrum* (Figs 32–33) slightly more slender, 1.15–1.40 × as long as pronotum.

*Pronotum* 1.02–1.18 × as wide as long.

*Elytra* 0.98–1.05 × as long as wide.

*Underside*. Ventrite V simple, lacking median sulcus or concavity.

*Terminalia and genitalia*. Sternite VIII (Figs 97, 107) with arms slender and apically divergent. Bursa copulatrix (Figs 95, 105) usually simple, but sometimes with dense minute coniform spicules in posterior part. Spermatheca (Figs 99, 109, 126, 128, 130, 132) with collum more strongly convex; insertions of duct and gland slightly more distant from each other.

**Figures 125–132.**
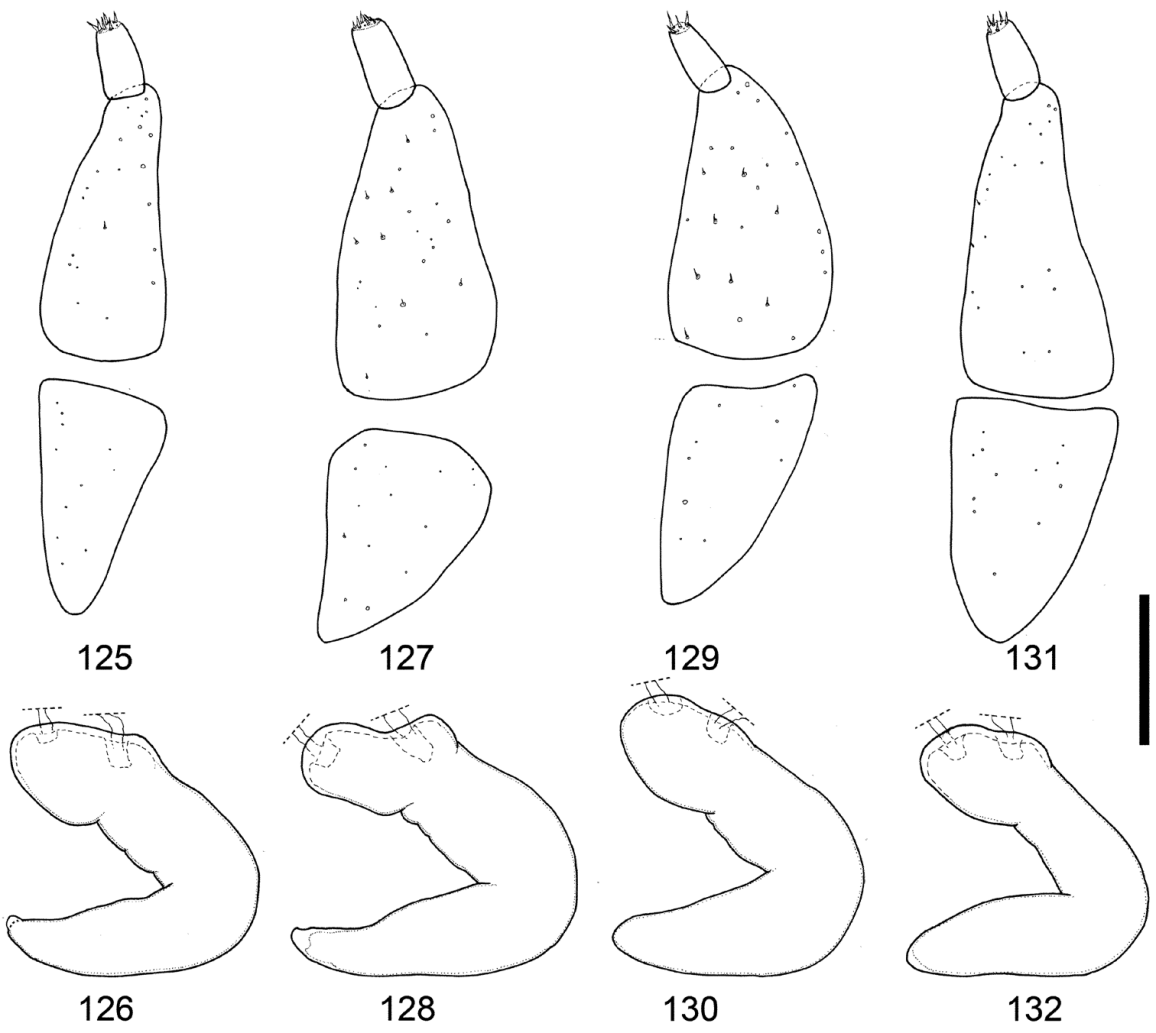
Female genitalia of *Scleropteroides horridulus* (Voss) from various localities. **125–126** Taiwan, China **127–128** Fujian, southeastern China **129–130** Yakushima Island, Nansei Islands, Japan **131, 132** Namwongun, South Korea. Scale bar: 0.10 mm.

Otherwise showing almost the same sexual dimorphism as in *Scleropteroides hypocrita*.

#### Material examined.

**CHINA: Hunan.** 1 male, Pingjiang, Mt. Mupushan, 1600 m, 1–12-VIII-2003, Li et al. (NIAES). **Fujian.** 2 males and 2 females, Jianyang, 27°20'N, 118°06'E, 18-IV-1965 (IZCAS, IOZ(E)896779, 896780, 896781, and 896786); 1 female, Jianyang, Aotou, Sanbanqiao, 27°35'N, 117°39'E, 18-IV-1965 (IZCAS IOZ(E)896788); 1 female, Jianyang, Aotou, Huangbaixi, 27°35'N, 117°39'E, 7-V-1965 (IZCAS IOZ(E)89679); 1 male, Jianyang, Aotou, Daoshui, 27°35'N, 117°39'E, 13-VI-1965 (IZCAS, IOZ(E)897690). 1 female, Shaowu, Tieyang, 27°10'N, 117°21'E, 15-V-1965 (IZCAS, IOZ(E)896789). 1 male, Wuyishan, Xingcun, Sangang, 752 m, 27°44.804'N, 117°40.600'E, 15-IV-2009, J. Huang (ZAFU). 1 female, Wuyishan, Xingcun, Sangang, 752 m, 27°44.804'N, 117°40.600'E, 15-IV-2009, S. Zhang (ZAFU). 1 male, Wuyishan, Xingcun, Qili, 899–912 m, 27°43.302'–27°43.042'N, 117°39.632'–117°39.334'E, 20-IV-2009, J. Tan (ZAFU). 1 female, Wuyishan, Xingcun, Qili, 899–912 m, 27°43.302'–27°43.042'N, 117°39.632'–117°39.334'E, 20-IV-2009, J. Huang (ZAFU). **Chongqing.** 1 female, Nanchuan, Yixiantian, 700 m, 29.05194°N, 107.12001°E, 12-VI-2010, R. Nie (IZCAS IOZ(E)1803625). **TAIWAN.** 1 male, Nantou, Sungkan–Meifeng, 25–26-V-1972, M. Sakai (EUMJ). 1 male and 1 female, Nantou, Nanshanchi, 20–23-III-1995, H. Kojima and M. Suehiro (NIAES). 1 male, Mt. Guandaoshan, 16-V-1986, I. Matoba (NIAES). 1 male, Taoyuan, Mt. Daguanshan, 4-IV-1991, H. Kojima (NIAES). **KOREA: Gangwondo.** 1 female, Chuncheongun, Dongmyeon, Gamjeongri, 21-V-1992, K. Morimoto (ELKU). **Kyeongsangnamdo.** 1 female, Mt. Jirisan, Samjeongri, 6-VI-1991, J. D. Bae (ELKU). **Jeollabukdo.** 1 male and 1 female, Namwongun, Nowondan, 5-VI-1991, J. D. Bae (ELKU). 1 female, Namwongun, Deongdongri, 19-VI-1994, H. Kojima (ELKU). **JAPAN: Nansei Islands.** YAKUSHIMA I. Nakama: 1 male, 1-IV-1985, A. Yoshida (NIAES); 1 male and 2 females, 25-III-1991, H. Kojima (ELKU); 4 males and 6 females, 30-III-2005, Y. Kubota (NIAES). AMAMI-OSHIMA I. 1 male, Sutaru-touge Pass, 23-III-1954, T. Mohri (EUMJ). Nishinakama: 1 female, 25-III-1954, T. Edashige (EUMJ); 1 female, 31-III-1968, M. Tomokuni (EUMJ); 1 male, 22-III-1990, T. Ueno (NIAES). Hatsuno: 1 male and 1 female, 24-III-1965, S. Fukuda (SM); 1 female, 12-IV-1971, M. Sakai (EUMJ); 1 female, 11-V-1976, J. Okuma (SM). 1 male, Daikuma, 10-IV-1971, M. Sakai (EUMJ). 1 female, Shinmura, 11-IV-1971, M. Sakai (EUMJ). Mt. Yuwandake: 1 male, 19-IV-1971, M. Sakai (EUMJ); 25 males and 18 females, 25–28-III-2003, H. Yoshitake, on *Rubus croceacanthus* (NIAES). 9 males and 8 females, Chuo-rindou, 18-III-1991, H. Kojima (ELKU). 2 males, Nangawa-rindou, 20-III-1991, H. Kojima (ELKU); 1 male and 2 females, 22-III-1991, H. Kojima (ELKU). 2 males and 5 females, Mt. Akatsuchiyama, 21-III-1991, H. Kojima (ELKU). UKEJIMA I. 1 male, 22-III-2004, K. Takahashi (NIAES). OKINAWAJIMA I. 1 female, Yona, 10-IV-1975, S. Imasaka (NIAES). 1 male, Oku, 15–18-V-1978, T. Tsutsumi (ELKU). 2 males and 1 female, Mt. Yonahadake, 16-IV-1991, H. Kojima (ELKU).

#### Distribution.

China (Chongqing and Fujian – new record, and Taiwan), Korea (Central and South – new record), Japan (Nansei Islands: Yakushima, Amami-Oshima, and Okinawajima Islands; Fig. 134).

#### Biological note.

A number of adults were collected from *Rubus croceacanthus* H.Lév. growing along the edge of an evergreen broad-leaved forest at the foot of Mt. Yuwandake, Amami-Oshima Island.

#### Comment.

In the study, we could not examine the holotype of *Rhytidosomus insularis* Voss, which should be preserved in ELKU, but K. Morimoto (pers. comm., 2005) had examined it and confirmed its taxonomic identity. Also, our examination of a long series of *Scleropteroides* specimens from the type locality, Amami-Oshima Island, revealed that only one species of this genus occurs on the island.

## Discussion

### Systematic position and definition of the genus

*Scleropteroides* was established as a genus allied to *Scleropterus* in the tribe Scleropterini (Colonnelli 1979). This placement was followed by subsequent authors (Alonso-Zarazaga and Lyal 1999, Hong et al.1999, Yoshitake et al. 2004, Colonnelli 2004, Korotyaev and Hong 2004), except by Morimoto (1989) who placed it in the tribe Ceutorhynchini. This is not unexpected as Scleropterini has never been clearly defined (Schultze 1902, Reitter 1913, Wagner 1937) and the morphological distinction between Scleropterini and Ceutorhynchini is still unclear because of inconsistencies in character states defining the two tribes (Colonnelli 1984, 2004). In addition, a recent molecular phylogenetic study (Kato et al. 2006) did not support the monophyly of either Ceutorhynchini or Scleropterini, although it supported a close relationship between *Scleropteroides* and *Scleropterus*. Here we follow the recent tribal classification by Colonnelli (2004) for convenience, although we acknowledge it is in need of revision.

Previously, *Scleropteroides* was defined by Colonnelli (1979, 2004) and Korotyaev (1996) as follows: 1) rostrum conspicuously expanded from the base to apex, wider than the profemur; 2) prothorax with large and extremely coarse punctures; 3) scutellar shield visible; 4) elytral humeri prominent; 5) all elytral intervals subequal in width and height; 6) each interval bearing acute squamate granules; 7) striae catenulate, very deep, at least as wide as intervals; 8) rostral channel extending to metaventrite; 9) femora dentate; and 10) claws dentate. However, this definition of *Scleropteroides* is inappropriate because it contained incorrectly described structures (1 and 7), as well as characteristics common to *Scleropterus* (2, 6, 8, and 10). Therefore, the diagnostic differences between the two genera must be clarified.

Morphologically, *Scleropteroides* is close to *Scleropterus* in sharing a rounded apex of the antennal scape, a six-segmented antennal funicle, a pronotum with neither tubercules nor prominences, elytral intervals bearing small and acute squamate granules usually in a row, a rostral channel extending to the metaventrite, and appendiculate claws. In addition, the close affinity between the two genera is emphasized by similarities in structures of the male and female genitalia.

On the basis of detailed morphological observations, we redefined *Scleropteroides* in this study as follows: 1) eyes convex (Figs 22–33); 2) antennal scape fringed with 3–4 linear scales at the apex, in addition to a few minute hairs; 3) basal margin of the pronotum bisinuate (Figs 10, 34, 36, 38); 4) pronotum lacking a sulcus (Figs 10, 34, 36, 38); 5) apical margin of the pronotum raised (Fig. 12); 6) scutellar shield well-developed, visible from above (Figs 11, 34–39); 7) hind wings well-developed (Fig. 19); 8) elytral humeri well-developed (Figs 11, 35, 37, 39); 9) elytral intervals subequal in width and height (Figs 11, 35, 37, 39); 10) femora dentate (Fig. 15); 11) protibiae simple, not incurved apically (Fig. 15); and 12) meso- and metatibiae mucronate only in males.

In contrast, *Scleropterus* has the following defining characteristics: 1) eyes flattened; 2) antennal scape fringed with 3–4 minute hairs at the apex; 3) basal margin of the pronotum nearly straight; 4) basal half of the pronotum with a weak longitudinal median sulcus; 5) apical margin of the pronotum slightly descending; 6) scutellar shield vestigial, usually invisible from above; 7) hind wings vestigial; 8) elytral humeri vestigial; 9) odd numbered intervals of the elytra wider and more prominent than even numbered intervals; 10) femora edentate; 11) protibiae incurved apically; and 12) meso- and metatibiae mucronate in both sexes.

The most apparent differences between the two genera are the structure of the hind wings (character 7) and associated characters (3, 6, and 8). Apart from these differences associated with hind-wing reduction in *Scleropterus*, however, *Scleropteroides* clearly differs from *Scleropterus* in having a pronotum lacking a sulcus, even elytral intervals, dentate femora, and simple protibiae. Additionally, *Scleropteroides* can also be distinguished from *Scleropterus* by the meso- and metatibiae lacking mucrones in females.

### Relationships among species

Our extensive and detailed examination of a large number of specimens revealed that *Scleropteroides* comprises three East Asian species – *Scleropteroides hypocrita*, *Scleropteroides horridulus*, and *Scleropteroides longiprocessus*. They are very similar in general appearance, as well as in general structures of male and female terminalia, but can be distinguished clearly by several consistent, taxonomically important morphological differences as shown in the following lines.

*Scleropteroides hypocrita* is characterized mainly by a moderately constricted prothorax in the subapical part (Fig. 34), gently convergent elytra toward the subapical calli (Fig. 35), moderately prominent humeri (Fig. 35), and elytral intervals with a row of semirecumbent scales that are evidently shorter than the interval width (Fig. 35). In addition, the species is also distinguished by sexual structures in males, such as a slightly widened rostrum in the apical half (Fig. 22), metaventrite that is weakly prominent along the apico-lateral margin of the metaventral receptacle, ventrite I bearing a median prominence along the apical margin, the penis with a blunt apical projection that is rounded at the apex (Figs 41, 51, 61), and a pair of fig-like sclerites on the basal part of the endophallus (Figs 40, 50, 60). In females, the arms of sternite VIII are apically arcuate (Figs 47, 57, 67) and the posterior part of the bursa copulatrix is densely covered with minute coniform spicules (Figs 45, 55, 65). The length of scales in a row on the elytral intervals is variable, from 0.4 to 0.8 times as long as the interval width, mostly ranging from 0.5 to 0.6 times as long. We examined specimens from various localities in detail, but no significant geographical variation was observed in the external or genital structures of either sex.

Colonnelli (2004) synonymized *Scleropteroides insularis* with *Scleropteroides hypocrita* in his world catalog of the subfamily Ceutorhynchinae; however, he gave no explanation for this taxonomic treatment. Our study revealed that *Scleropteroides insularis* is actually identical with *Scleropteroides horridulus*. *Scleropteroides horridulus* shows remarkable morphological differences from *Scleropteroides hypocrita* in the strongly constricted subapical part of the prothorax (Fig. 38), straightly convergent elytra toward the subapical calli (Fig. 39), strongly prominent humeri (Fig. 39), erect scales on elytral intervals that are much longer than the interval width (Fig. 39), and ventrite I lacking a median prominence. In addition, *Scleropteroides horridulus* clearly differs from *Scleropteroides hypocrita* in having the following male sexual traits: metaventrite more strongly prominent ventrally along the apico-lateral margin of the metaventral receptacle, apical projection of the penis truncate at the apex (Figs 91, 101, 111, 114, 117, 120, 123), and paired sclerites in the basal part of the endophallus weakly falcate (Figs 95, 105). Furthermore, *Scleropteroides horridulus* has apically divergent arms on sternite VIII in females (Figs 97, 107). We consider *Scleropteroides horridulus* a valid species despite of Colonnelli’s (2004) synonymy of *Scleropteroides insularis* with *Scleropteroides hypocrita*. This species shows geographical variation in female genital structure. In specimens from continental China and Korea, the bursa copulatrix is densely armed with minute spicules in the posterior part (Fig. 105), whereas it is unarmed in specimens from Taiwan and the Nansei Islands (Fig. 95). This may suggest the presence of a cryptic species because structures of the bursa copulatrix are usually conservative and not variable within a species.

*Scleropteroides longiprocessus* shows character states intermediate between *Scleropteroides hypocrita* and *Scleropteroides horridulus* in having a strong constriction in the subapical part of the prothorax (Fig. 36), strongly prominent humeral calli (Fig. 37), and elytral intervals with semierect scales, each of which is nearly as long as the interval width (Fig. 37). This species was previously confused with *Scleropteroides hypocrita* due to their close resemblance in general appearance and the lack of a detailed morphological examination. However, *Scleropteroides longiprocessus* differs from *Scleropteroides hypocrita* by having a stouter rostrum (Fig. 26), more rapidly convergent elytra toward the subapical calli (Fig. 37), and ventrite I lacking a median prominence. Moreover, *Scleropteroides longiprocessus* is distinguished by male sexual traits, such as a metaventrite that is more prominent ventrally along the apico-lateral margin of the metaventral receptacle, an elongate projection at the apex of the penis (Figs 71, 81), and the paired falcate sclerites in the basal part of the endophallus (Figs 70, 80). With regard to female sexual traits in *Scleropteroides longiprocessus*, the arms of sternite VIII are apically divergent (Figs 77, 87) and the bursa copulatrix lacks spicules (Figs 75, 85). This species shows non-geographical intraspecific variation in the length of scales on the elytral intervals. Most specimens examined had scales slightly longer than the interval width, but some specimens bore stouter scales that were slightly shorter than the interval width.

Additionally, *Scleropteroides longiprocessus* resembles *Scleropteroides horridulus* in having straightly convergent elytra toward the subapical calli (Figs 37, 39), a metaventrite that is moderately prominent ventrally along the apico-lateral margin of the metaventral receptacle in males, ventrite I lacking a median prominence in both sexes, and the apically divergent arms of female sternite VIII (Figs 77, 87, 97, 107). However, *Scleropteroides longiprocessus* is easily distinguished from *Scleropteroides horridulus* by the more strongly widened rostrum in males (Fig. 26), the pronotum with subparallel sides in the basal half and a weaker subapical constriction, less-developed elytral humeri, less erect and shorter scales on the elytral intervals (Fig. 37), a penis with a slender apical projection that is rounded at the apex (Figs 71, 81), narrower sclerites in the basal part of the endophallus (Figs 70, 80), and an unarmed bursa copulatrix (Figs 75, 85).

The affinity of the three *Scleropteroides* species is still uncertain and a phylogenetic analysis of the genus is necessary to clarify their relationships.

### Host plant associations

*Rubus* is a cosmopolitan genus and contains an estimated 900–1000 species worldwide (Thompson 1997). *Rubus* are shrubs or subshrubs, deciduous, rarely evergreen or semievergreen, and sometimes perennial creeping dwarf herbs (Lu and Boufford 2003). To date, 249 *Rubus* species have been recorded from China (including Taiwan), Korea, and Japan (Lu and Boufford 2003, KPNI 2008, Yonekura and Kajita 2003). In East Asia, *Rubus* species constitute the understory of open woodlands, especially in and around clearings and margins. They also occur in more open, sunny locations along forest roads and along rivers.

Presently, ecological information on *Scleropteroides* weevils is limited and their host plant associations are not clear (Table 2). However, the available data indicate that *Scleropteroides* weevils are associated with woody *Rubus* species belonging to the subgenus *Idaeobatus* and growing in and around woodlands at least in the adult stage because adults were collected from *Rubus* shrubs and observed feeding on leaves and flowers of the plants on many occasions. With regard to adult feeding habits, *Scleropteroides hypocrita* widely utilizes at least four *Rubus* species (*Rubus crataegifolius*, *Rubus idaeus* subsp. *melanolasius* f. *concolor*, *Rubus microphyllus*, and *Rubus trifidus*) in Korea and Japan, whereas *Scleropteroides longiprocessus* was observed feeding on *Rubus palmatus* in Honshu and Kyushu, and *Scleropteroides horridulus* on *Rubus croceacanthus* in the Ryukyus. In two localities in Toyota City, Honshu, Japan, adults of *Scleropteroides hypocrita* and *Scleropteroides longiprocessus* were found separately on *Rubus microphyllus* and *Rubus palmatus* var. *coptophyllus*, respectively (K. Izawa, pers. comm., 2014). Similarly, on Mt. Kusenbuyama, Kyushu, Japan, adults of *Scleropteroides hypocrita* and *Scleropteroides longiprocessus* were collected separately on *Rubus crataegifolius* and *Rubus palmatus*, respectively, except a few individuals of *Scleropteroides longiprocessus* collected simultaneously with *Scleropteroides hypocrita* on *Rubus crataegifolius*. These observations suggest that *Scleropteroides* species might show some differences in host use in localities where they occur sympatrically. As mentioned above, *Scleropteroides hypocrita* is sympatric with *Scleropteroides longiprocessus* in Japan and with *Scleropteroides horridulus* in South Korea (Table 1). Further studies are needed to explore the host plant range of each *Scleropteroides* species, especially in sympatric localities.

**Table 1.** Localities where *Scleropteroides* species occur sympatrically.

Species	Locality
*Scleropteroides hypocrita* and *Scleropteroides horridulus*	IN THE KOREAN PENINSULA:
	GANGWONDO: Chuncheongun, Dongmyeon, Gamjeongri
	KYEONGSANGNAMDO: Mt. Jirisan, Samjeongri
	JEOLLABUKDO: Namwongun, Deongdongri
*Scleropteroides hypocrita* and *Scleropteroides longiprocessus*	IN THE JAPANESE ARCHIPELAGO:
	IWATE: Kawai, Tsuchisaka-touge
	AKITA: Lake Tazawako
	YAMAGATA: Mt. Zaousan, Yoshikari-rindou
	FUKUSHIMA: Haranomachi, Kozikiishi-rindou; Namie
	IBARAKI: Yamizosan, Shimokitazawa
	TOCHIGI: Fujiwara, Midorizawa-rindou
	GUMMA: Matsuida, Kirizumi-onsen
	SAITAMA: Shoumaru-touge Pass
	TOKYO: Okutama, Kurasawadani; Mt. Oodakeyama; Mt. Takaosan
	KANAGAWA: Tanzawa Mts., Yabitsu-touge Pass; Tanzawa Mts., Mizunashigawa; Yamakita, Mikuni-touge Pass
	CHIBA: Mt. Kiyosumiyama–Kenminnomori
	NAGANO: Nakakawa; Tobira-onsen
	SHIZUOKA: Shinfuji
	AICHI: Toyota, Nishiichinonochou; Toyota, Asugawachou, Asugawa-hoan-rindou
	MIE: Misugi, Hirakura
	SHIGA: Mt. Ibukiyama
	FUKUOKA: Mt. Hikosan
	NAGASAKI: Tashirobaru; Sasebo, Mt. Hattendake

**Table 2.** Summary of plant association data for *Scleropteroides*.

Species	Host plant	Observation*	Locality	Author
*Scleropteroides hypocrita*	*Rubus* spp.	A	Japan	Morimoto (1984)
*Scleropteroides hypocrita*	*Rubus crataegifolius*	A	Japan, Honshu: Mt. Takaosan	This study
*Scleropteroides hypocrita*	*Rubus crataegifolius*	A	Japan, Honshu: Iwate	This study
*Scleropteroides hypocrita*	*Rubus crataegifolius*	A	Japan, Kyushu: Mt. Kusenbuyama	This study
*Scleropteroides hypocrita*	*Rubus* sp.	A	Japan, Honshu: Fukushima	This study
*Scleropteroides hypocrita*	*Rubus trifidus*	A	Japan, Honshu: Gifu, Toki	This study
*Scleropteroides hypocrita*	*Rubus trifidus*	A	Japan, Honshu: Aichi, Toyota, Oobora, Nikake-rindou	This study
*Scleropteroides hypocrita*	*Rubus microphyllus*	A	Japan, Honshu: Aichi, Asugawachou, Asugawa-hoan-rindou	This study
*Scleropteroides hypocrita*	*Rubus microphyllus*	A	Japan, Honshu: Mie, Komonochou, Komono	This study
*Scleropteroides hypocrita*	*Rubus idaeus* subsp. *melanolasius* f. *concolor*	A	Korea	Hong et al. (1999), Korotyaev and Hong (2004)
*Scleropteroides hypocrita*	*Rubus crataegifolius*	A	Korea, South: Mt. Jirisan	This study
*Scleropteroides longiprocessus*	*Rubus palmatus*	A	Japan, Kyushu: Mt. Kusenbuyama	This study
*Scleropteroides longiprocessus*	*Rubus palmatus* var. *coptophyllus*	A	Japan, Honshu: Tokyo, Hachiouji, Mt. Takaosan	This study
*Scleropteroides longiprocessus*	*Rubus palmatus* var. *coptophyllus*	A	Japan, Honshu: Gifu, Nakatsugawa, Nenoue-kougen	This study
*Scleropteroides longiprocessus*	*Rubus palmatus* var. *coptophyllus*	A	Japan, Honshu, Aichi: Shitara, Mt. Nishinagura-Iyama	This study
*Scleropteroides longiprocessus*	*Rubus palmatus* var. *coptophyllus*	A	Japan, Honshu, Aichi, Toyota: Nishiichinonochou; Asugawachou, Asugawa-hoan-rindou; Inabuchou, Noirigawa	This study
*Scleropteroides horridulus*	*Rubus croceacanthus*	A	Japan, Ryukyus: Amami-Oshima I.	This study

* A: Adults, feeding behavior was confirmed.

Generally, each species in the subfamily Ceutorhynchinae utilizes the same host plant in the adult and larval stages (e.g., Hoffmann 1954, Jordheuil 1963, Dieckmann 1972). Therefore, *Scleropteroides* weevils would be suspected to be associated with *Rubus* species in their larval stage. *Scleropteroides* adults appear before and during the flowering season of *Rubus* and were frequently found on reproductive organs of the plants. Further surveys focusing on larval food sources are necessary to elucidate the host plant associations of *Scleropteroides* weevils.

### Distribution and biogeography

**Figure 133.**
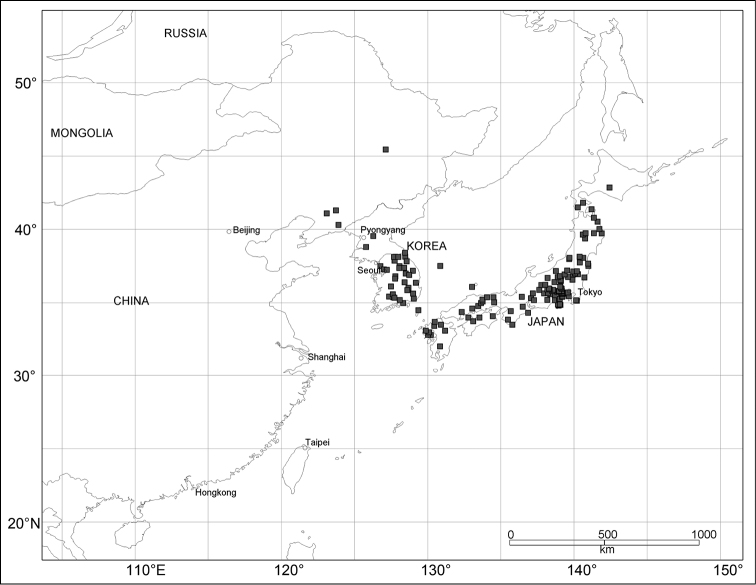
Geographic distribution of *Scleropteroides hypocrita* (Hustache) (■).

**Figure 134.**
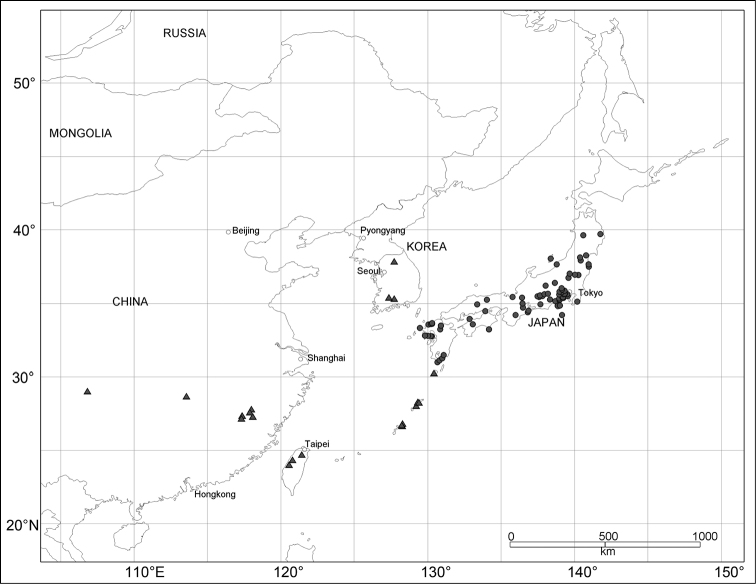
Geographic distributions of *Scleropteroides longiprocessus* Huang & Yoshitake, sp. n. (●) and *Scleropteroides horridulus* (Voss) (▲).

The distribution of *Scleropteroides* is limited to East Asia (Figs 133–134), in contrast to *Scleropterus*, which is widely distributed from Central Europe through Central Asia to East Asia (Colonnelli 2004).

**Figures. 135–138.**
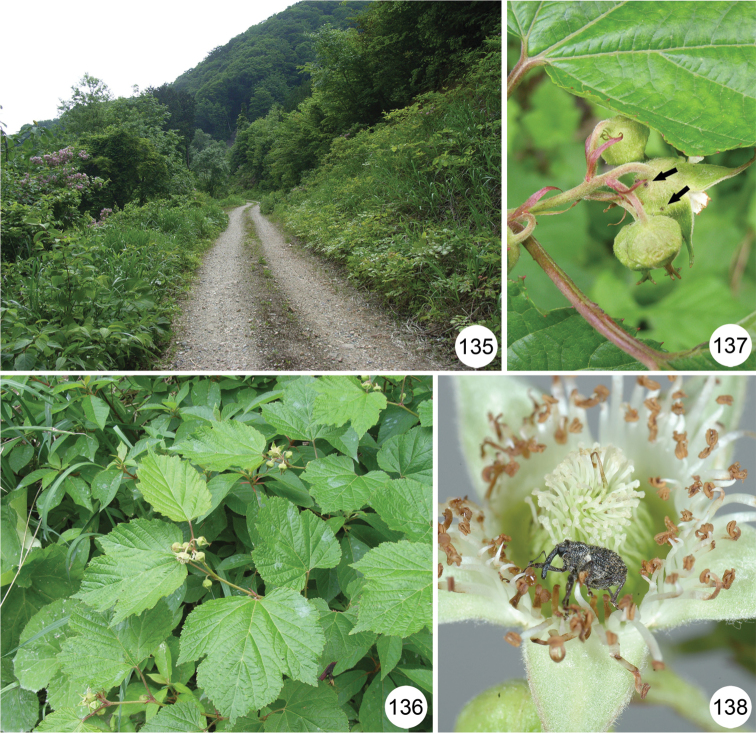
*Scleropteroides hypocrita* (Hustache). **135** Habitat in Toyosawa-rindou, Iwate, Honshu, Japan **136** Adult food plant, *Rubus crataegifolius* **137** Feeding traces of adults on a flower bud of *Rubus crataegifolius* (indicated with arrows) **138** Adult on a flower of *Rubus crataegifolius*.

*Scleropteroides hypocrita* is distributed in northeast China, North and South Korea, and Japan (Fig. 133), whereas *Scleropteroides longiprocessus* is restricted to Japan (Fig. 134). The two species occur sympatrically in many localities in Japan (Table 1). Further, *Scleropteroides horridulus* is distributed from Fujian through Taiwan to the Nansei Islands and in South Korea (Figs 133–134). This species is sympatric with *Scleropteroides hypocrita* at least in the Korean Peninsula (Table 1), but the distributional boundary of the two species is still unclear in continental China.

Currently, a considerable gap still remains in the distribution of *Scleropteroides*, especially in continental China and the Korean Peninsula. In addition, this genus has never been recorded from the southern part of the Russian Far East. Since the distribution of *Scleropteroides* strongly suggests its occurrence in these regions, further surveys are necessary to elucidate the range of the genus and that of each species.

### Key to species

**Table:** 

1	Subapical part of the prothorax moderately constricted (Fig. 34). Elytra gently convergent toward the subapical calli, with weakly prominent humeri. Scales on the elytral intervals semirecumbent, evidently shorter than the interval width (Fig. 35). Metaventrite weakly prominent ventrally along the apico-lateral margin of the metaventral receptacle in males. Ventrite I with a median prominence along the apical margin in males. Female sternite VIII with apically arcuate arms (Figs 47, 57, 67). [Rostrum slightly widened in the apical half in males. Male genitalia with a blunt projection at the apex of the penis (Figs 41, 51, 61) and a pair of fig-like sclerites in the basal part of the endophallus (Figs 40, 50, 60). Female genitalia with the bursa copulatrix bearing dense, minute, coniform spicules in the posterior part (Figs 45, 55, 65).]	*Scleropteroides hypocrita*
–	Subapical part of the prothorax strongly constricted (Figs 36, 38). Elytra straightly convergent toward the subapical calli, with strongly prominent humeri. Scales on elytral intervals more or less erect, evidently longer than or nearly as long as the interval width (Figs 37, 39). Metaventrite more strongly prominent ventrally along the apico-lateral margin of the metaventral receptacle in males. Ventrite I simple, lacking a median prominence in both sexes. Female sternite VIII with arms divergent apically (Figs 77, 87, 97, 107)	2
2	Apical half of the rostrum slightly widened in males (Fig. 30). Pronotum subparallel-sided in the basal 2/3, with a strong subapical constriction. Elytral humeri strongly prominent. Scales on elytral intervals erect, much longer than the width of each interval (Fig. 39). Apical projection of the penis blunt, truncate at the apex (Figs 91, 101, 111, 114, 117, 120, 123). Basal part of the endophallus with a pair of weakly falcate sclerites. Bursa copulatrix simple (Fig. 95) or with minute coniform spicules in the posterior part (Fig. 105)	*Scleropteroides horridulus*
–	Rostrum more strongly widened in the apical half in males (Fig. 26). Basal half of the pronotum subparallel-sided, with a weaker subapical constriction. Elytral humeri slightly less prominent. Scales on elytral intervals semierect, nearly as long as the interval width (Fig. 37). Apical projection of the penis sharp, rounded at the apex (Figs 71, 81). Basal part of the endophallus with a pair of narrow falcate sclerites (Figs 70, 80). Bursa copulatrix simple, lacking spicules (Figs 75, 85)	*Scleropteroides longiprocessus*

## Supplementary Material

XML Treatment for Scleropteroides

XML Treatment for Scleropteroides hypocrita

XML Treatment for Scleropteroides longiprocessus

XML Treatment for Scleropteroides horridulus
